# The landscape of gene loss and missense variation across the mammalian tree informs on gene essentiality

**DOI:** 10.1038/s41588-026-02783-9

**Published:** 2026-10-20

**Authors:** Calwing Liao, Robert Ye, Franjo Ivankovic, Jack M. Fu, Raymond Walters, Chelsea Lowther, Elise Valkanas, Claire Churchhouse, Kaitlin E. Samocha, Kerstin Lindblad-Toh, Elinor Karlsson, Michael Hiller, Michael E. Talkowski, Benjamin M. Neale

**Affiliations:** 1 Analytic and Translational Genetics Unit, Department of Medicine, Massachusetts General Hospital, Boston, MA USA; 2 Stanley Center for Psychiatric Research, Broad Institute of MIT and Harvard, Cambridge, MA USA; 3 Center for Genomic Medicine, Massachusetts General Hospital, Boston, MA USA; 4 Science for Life Laboratory, Uppsala University, Uppsala, Sweden; 5 Genomics and Computational Biology, UMass Chan Medical School, Worcester, MA USA; 6 Program in Molecular Medicine, UMass Chan Medical School, Worcester, MA USA; 7 LOEWE Centre for Translational Biodiversity Genomics, Frankfurt, Germany; 8 Senckenberg Research Institute, Frankfurt, Germany; 9 Institute of Cell Biology and Neuroscience, Faculty of Biosciences, Goethe University Frankfurt, Frankfurt, Germany

**Keywords:** Genetics, Genomics

## Abstract

Patterns of gene and sequence preservation across species provide insight into gene essentiality under natural selection. Here we developed two interspecies constraint metrics across 462 mammalian species: Gene Identity Score of Mammalian Orthologs (GISMO), which quantifies gene loss weighted by evolutionary distance to humans, and GISMO-mis, which measures missense constraint using the ratio of missense to synonymous variation across mammals. Both metrics are strongly correlated with human loss of function, missense and dosage constraint derived from large-scale sequencing studies, yet are largely independent of coding sequence length. GISMO and GISMO-mis identify essential genes missed by existing human-only metrics, particularly short genes. Genes in the most constrained deciles are enriched for rare variant associations in disorders linked to reduced fecundity, including schizophrenia, autism and neurodevelopmental disorders, and show strong enrichment for common variant heritability. These deciles also exhibit reduced copy number variation, consistent with dosage sensitivity. In addition, GISMO improves prioritization of recessive disease genes and captures signatures of homozygous selection across the mammalian tree. Taken together, GISMO and GISMO-mis leverage deep mammalian evolution to provide complementary, length-robust measures of gene essentiality that strengthen disease–gene prioritization and illuminate the selective forces shaping human biology.

## Abstract (web summary)

GISMO and GISMO-mis are new metrics of evolutionary constraint developed across 462 mammalian species. These metrics provide complementary measures of gene essentiality and improve prioritization of human disease genes.

## Main

The application of sequencing technologies at scale has enabled the quantification of gene essentiality in humans, particularly for loss-of-function (LoF) and missense genetic variants^1,2^. From an evolutionary perspective, genes vary in LoF tolerance and conservation. Genes that are essential for survival, development and reproduction tend to be more constrained and as damaging, and LoF variants in these genes lower fitness and often have severe detrimental effects^3,4^.

Measuring the selective constraint in humans has been made possible by large-scale genomic datasets such as the Genome Aggregation Database (gnomAD)^2^. The gnomAD consortium catalogs variants across >800,000 individuals and metrics such as LoF observed or expected upper bound fraction (LOEUF) and most observed or expected upper bound fraction (MOEUF) compare observed LoF and missense variation with mutation-rate-calibrated expectations. These variants in functionally important genes are often depleted because they are less likely to propagate across generations^5^.

The use of human constraint metrics has already provided valuable insights into the genetic basis of diseases and has been transformative for clinical genetics^6,7^. Genes associated with human genetic disorders that reduce fecundity, such as developmental disorders, are often found to have low LOEUF scores, indicating that these genes carry substantial consequences from a natural selection perspective^8–11^.

One of the main limitations of LOEUF is that genes with shorter sequences have fewer potential LoF mutation sites and attempts to quantify the reduction in such mutations is frustrated by the limited number of potential mutations (that is, it is difficult to determine whether the number of sites observed is low when the expectation for the number of sites itself is low)^2^. Furthermore, quantification of intraspecies genetic variation requires vastly large sample sizes and can be costly^2,12^. One potential solution to address these limitations is to leverage evolutionary conservation, a principle long recognized for identifying functionally important variants. Foundational methods such as scale-invariant feature transform (SIFT) and subsequent variant-centric tools like PolyPhen, MutPred, combined annotation-dependent depletion (CADD) and rare exome variant ensemble learner (REVEL) have consistently demonstrated the utility of conservation across species in predicting variant pathogenicity^13–17^.

Orthologs descend from a common ancestral gene through speciation and often retain similar sequences and functions across species^18^. Characterization of orthologs therefore informs evolutionary relationships and functional conservation across organisms. This is particularly true in mammals where the study of orthologs has enabled the investigation of the genetic basis of various biological processes and diseases^19^. Although mammals are physiologically and anatomically diverse^20,21^, their shared ancestry allows ortholog comparisons to reveal evolutionary changes shaping mammalian biology^22^. Comparative genomics studies have shown that orthologs often retain similar functions, suggesting a strong selective pressure to maintain their essential roles throughout evolution^18,23,24^.

Here we characterized gene loss and missense changes across placental mammalian reference genomes and developed Gene Identity Score of Mammalian Orthologs (GISMO), a gene-conservation metric spanning 462 species or ~10% of recognized placental mammals. We also developed GISMO-mis, which measures the missense-to-synonymous ratio of each gene. Both metrics correlate with human LoF, missense and dosage-sensitivity measures while aggregating evolutionary signals across entire genes. By operating across longer timescales, GISMO identifies short essential genes that may be missed by intraspecies metrics such as LOEUF. GISMO also captures common variant heritability and rare variant associations for traits with reduced fecundity, including neurodevelopmental disorders, and prioritizes recessive disease genes by capturing both heterozygous and homozygous selection. Overall, our findings will contribute to understanding the intrinsic properties of genes, the maintenance of essential gene functions and gene prioritization for human disease.

## Results

### Characterization of gene loss, missense and synonymous variation across mammals

To characterize gene loss, missense and synonymous variation, we analyzed orthologs from 462 placental mammalian species. Using human gene annotations as the reference, we inferred mammalian orthologs with the tool to infer orthologs from genome alignments (TOGA)^25^. TOGA codon alignments identified synonymous, missense and gene-inactivating mutations, including frameshifts, stop codons, splice-site mutations and larger deletions. We defined gene loss as the absence of an intact reading frame in other mammals, although the dataset includes human-specific or primate-specific genes absent outside primates. For example, 12 genes have been lost across all species except humans, which were typically pseudogenes or single-exon genes (Supplementary Table 1).

**Fig. 1:**
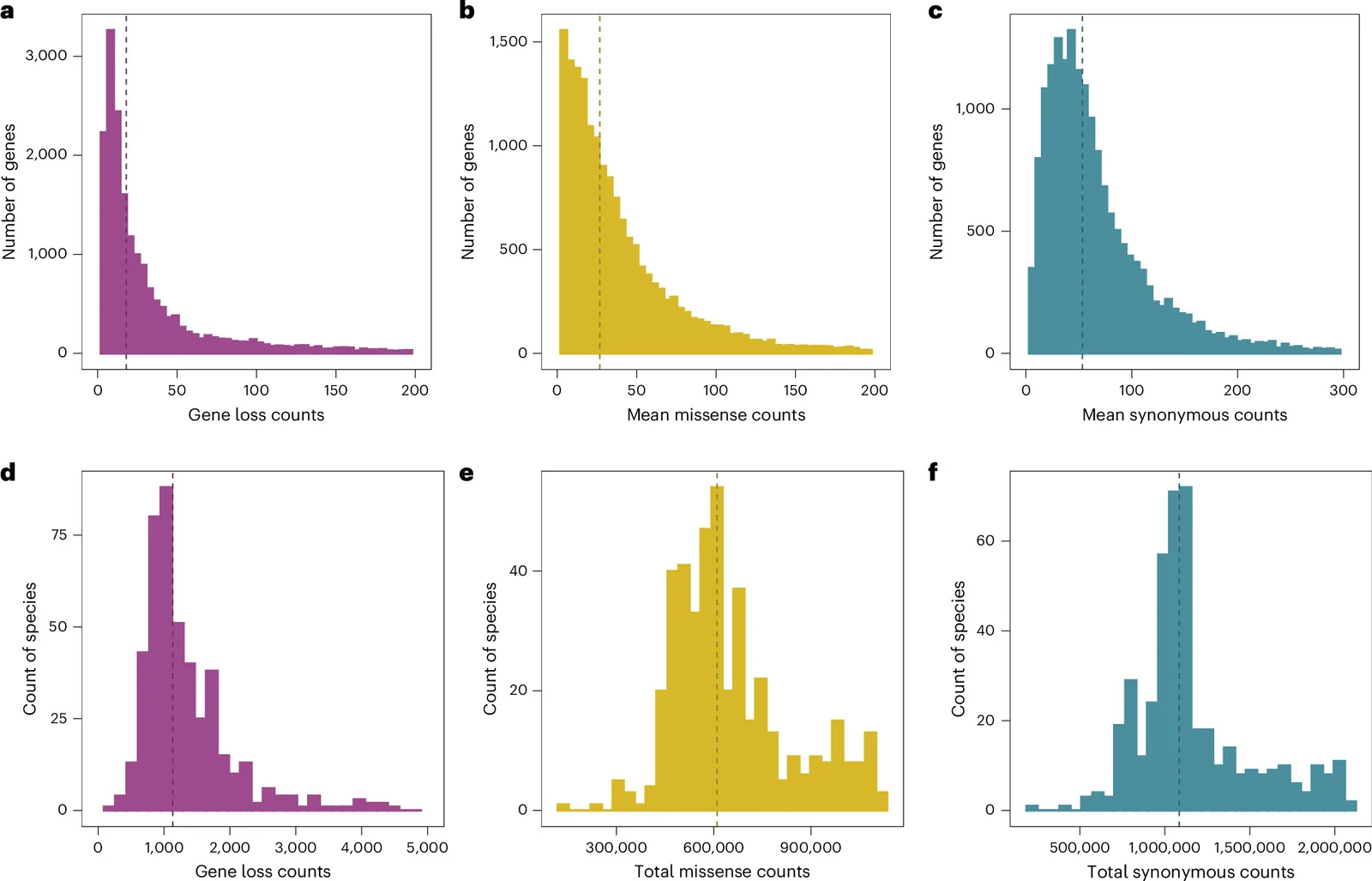
Characterization of gene loss, missense and synonymous counts across 462 placental mammals. **a**, Distribution of gene loss. The dashed vertical line indicates the median gene loss count. **b**, Mean missense variant counts across genes relative to the mammalian consensus. The dashed vertical line indicates the median number of missense counts relative to the mammalian consensus sequence. **c**, Mean synonymous variant counts across genes relative to the mammalian consensus. The dashed vertical line indicates the median number of synonymous counts relative to the mammalian consensus sequence. **d**, Frequency of gene loss per species. The dashed vertical line indicates the median count of gene loss events per species. **e**, Total missense variant counts per species. The dashed vertical line indicates the median total number of missense counts per species relative to the mammalian consensus sequence. **f**, Total synonymous variant counts per species. The dashed vertical line indicates the median total number of synonymous counts per species relative to the mammalian consensus sequence.

We found that each gene had a median of 18 gene loss events (Fig. 1a), whereas, across the species, we found a median of 1,130 likely gene loss events per species (Fig. 1d). Here we defined gene loss as disruption of the open reading frame. We identified only 6 genes with no instances of gene loss across the 462 mammalian species: *NAP1L1*, *ZC3H14*, *MEIS2*, *STRIP1*, *CCT5* and *ATP6V0D1*.

For each gene, we calculated the mean missense and synonymous count across the mammals. The median missense average was 27.7 (Fig. 1b), whereas the median synonymous average was 53.4 (Fig. 1c). For each species, there was a median of 610,603 missense variants (Figs. 1e) and 1,084,983 synonymous variants (Fig. 1f) across all genes.

### Defining GISMO and GISMO-mis

To capture human gene essentiality, we developed GISMO, which estimates the proportion of gene loss across 462 mammals, weighted by evolutionary distance from humans. Lower GISMO values indicate less frequent mammalian gene loss (Supplementary Table 2). We assessed the effects of phylogenetic compared to nonphylogenetic gene loss counts and found that, in general, there was a strong correlation between both (*R* = 0.95, *P* < 1 × 10^−16^; Supplementary Fig. 1). In addition, we assessed the effects of forward and reverse weighing of evolutionary distance and found that upweighing gene loss events that occurred more distant from humans led to empirically stronger results over conservation and constraint metrics (Supplementary Fig. 2). In particular, we found that GISMO and GISMO-mis were more strongly associated with other human constraint metrics than other conservation metrics such as mean coding sequences (CDS) genomic evolutionary rate profiling scores (Supplementary Fig. 2). We released the phylogenetic version of GISMO in Supplementary Table 11, but have moved forward with all analyses with the regular GISMO due to stronger associations with other constraint metrics. We also quantified missense differences relative to a mammalian consensus sequence as a proxy for molecular adaptation and selection. GISMO-mis measures the mean mammalian missense-to-synonymous ratio (Supplementary Table 3). We benchmarked the number of genes with a score compared to other metrics and found that GISMO covered the highest proportion at 97.7% of all genes in GENCODE v38 (Supplementary Table 4).

**Fig. 2:**
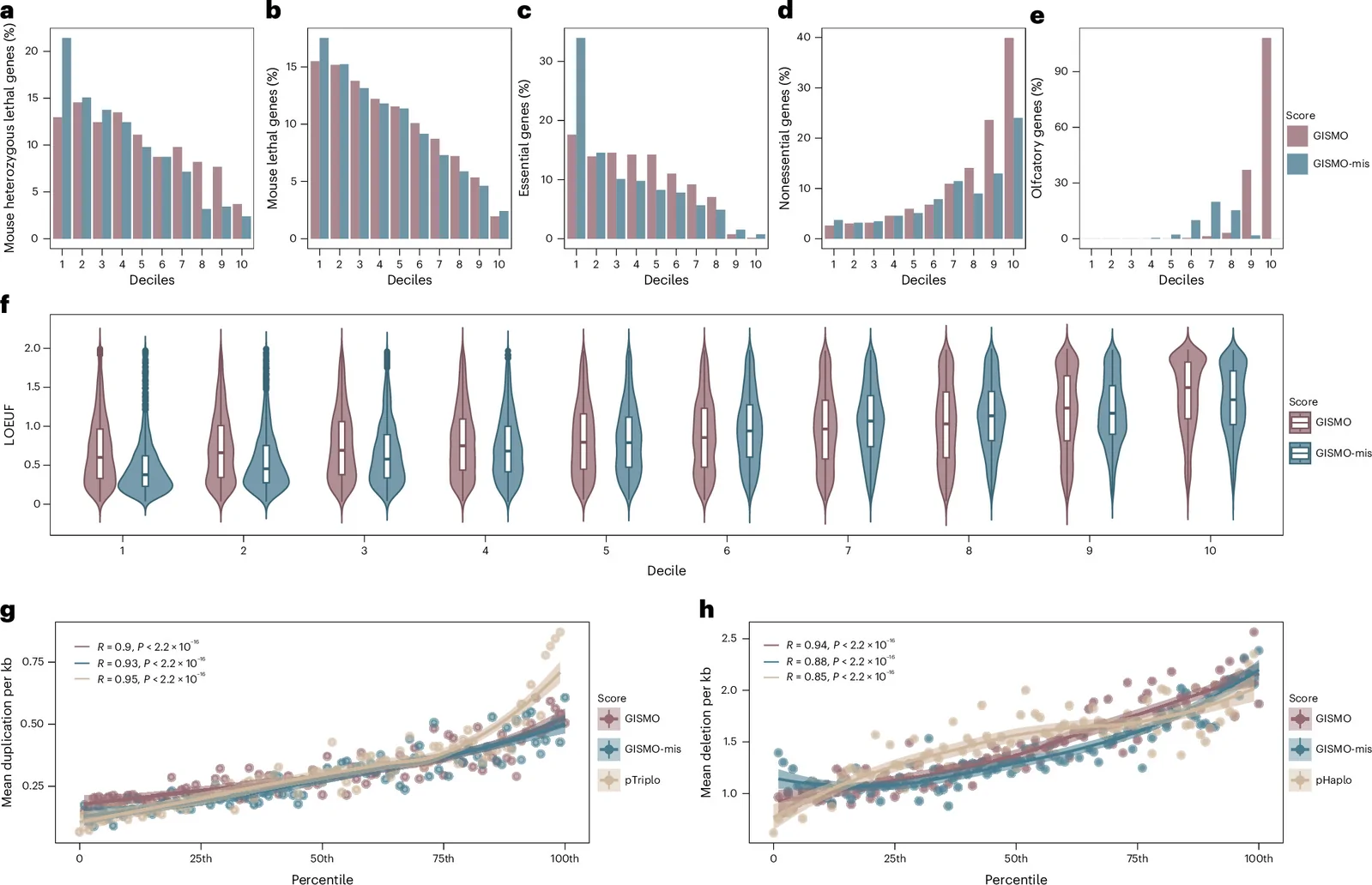
The biological and functional spectrum of gene loss and fixed missense differences. **a**–**e**, The percentage of genes present in each decile of GISMO and GISMO-mis across different gene sets (mouse heterozygous lethal (**a**), mouse lethal (**b**), essential (**c**), nonessential (**d**) and olfactory (**e**) genes). The first decile indicates the most constrained one. **f**, Comparison of GISMO and GISMO-mis to human LoF constraint. Violin plots show the distribution of values within each conservation-score decile. Embedded box plots indicate the median and interquartile range (IQR), with whiskers extending to 1.5× the IQR. The white circles indicate the mean. **g**,**h**, Comparison of GISMO and GISMO-mis to CNVs in the UK Biobank: duplications (**g**) and deletions (**h**). For pHaplo and pTriplo, the score was inverted (1 − pTriplo; 1 − pHaplo) such that the lowest percentiles indicated the most constrained to be consistent with other constraint metrics. A Spearman’s correlation test was done for each constraint and conservation score against the mean deletion or duplication per kilobase. The lines were estimated by a locally estimated scatterplot smoothing curve with a 95% CI.

We evaluated both metrics against other measures of biological and functional importance of genes. The most constrained GISMO and GISMO-mis deciles had the highest enrichment of genes that cause lethality in mouse heterozygous knockouts (odds ratio (OR)_GISMO_ = 3.43 (95% confidence interval (CI) 2.70–4.33), *P* = 1.68 × 10^−21^; OR_GISMO-mis_ = 5.78 (4.61–7.21), *P* = 2.93 × 10^−46^), mouse homozygous knockouts (OR_GISMO_ = 8.21 (95% CI 6.42–9.08), *P* < 1 × 10^−300^; OR_GISMO-mis_ = 13.1 (11.70–14.70), *P* < 1 × 10^−300^) and considered essential in cell lines (OR_GISMO_ = 4.34 (95% CI 3.64–5.17), *P* = 3.48 × 10^−52^; OR_GISMO-mis_ = 10.70 (9.05–12.70), *P* = 6.03 × 10^−152^) (Fig. 2a–c). The least constrained deciles have the highest proportion of nonessential and olfactory genes (Fig. 2d,e). We also found that genes that were frequently lost across the evolutionary tree were expressed in fewer tissues across Genotype-Tissue Expression (GTEx; Supplementary Fig. 3). We additionally assessed whether GISMO had any biases toward gene family size. We found that the distributions did not drift as family size increased (Supplementary Fig. 4).

We compared both metrics against human constraint metrics and found that GISMO (*R* = 0.38, *P* < 1 × 10^−300^) and GISMO-mis (*R* = 0.56, *P* < 1 × 10^−300^) were both strongly associated with LOEUF (Fig. 2f and Supplementary Fig. 5), with GISMO-mis having a stronger association. Both metrics also had strong correlations with missense constraint (MOEUF), which was comparable to LOEUF for GISMO-mis (*R* = 0.61, *P* < 1 × 10^−300^) but lower for GISMO (*R* = 0.33, *P* < 1 × 10^−300^). In addition, we found that both metrics were strongly associated with dosage sensitivity metrics to predict the strength of selection against loss of a gene copy (haploinsufficiency (pHaplo); *R*_GISMO_ = − 0.32, *P* < 1 × 10^−300^; *R*_GISMO-mis_ = −0.44, *P* < 1 × 10^−300^) and duplication of a gene (triplosensitivity (pTriplo); *R*_GISMO_ = − 0.32, *P* < 1 × 10^−300^; *R*_GISMO-mis_ = −0.50, *P* < 1 × 10^−300^). In addition, we compared GISMO-mis with the new gene-level AlphaMissense metric and found a stronger correlation to this metric (*R* = 0.84, *P* < 1 × 10^−300^) than LOEUF or missense constraint. We also compared the recently developed sHet metric against GISMO and GISMO-mis, which was developed to account for shorter gene bias. We found that both metrics had strong correlations, albeit weaker for GISMO, consistent with GISMO capturing homozygous selection (*R*_GISMO_ = −0.28, *P* < 1 × 10^−300^; *R*_GISMO-mis_ = −0.61, *P* < 1 × 10^−300^). Finally, we compared against conservation metrics such as mean genomic evolutionary rate profiling and mean phylogenetic codon substitution frequencies and found that GISMO and GISMO-mis consistently outperformed in terms of association strength with other constraint metrics relative to previous conservation scores (Supplementary Fig. 2).

Next, to assess the relationship between copy number variants (CNVs) and mammalian gene conservation, we leveraged a CNV dataset generated from exome sequencing in the UK Biobank^26^ (*n* = 197,306) that enabled detection of individual gene-level variants. The most constrained deciles of both GISMO and GISMO-mis had the lowest number of deletions and duplications. We found that GISMO had an even stronger correlation in the UK Biobank for deletions compared to pHaplo, a dosage-sensitivity metric derived from CNVs aggregated from lower-resolution microarray data across ~1 million human samples^27^ (Fig. 2g,h).

### Leveraging GISMO to identify shorter intolerant genes

**Fig. 3:**
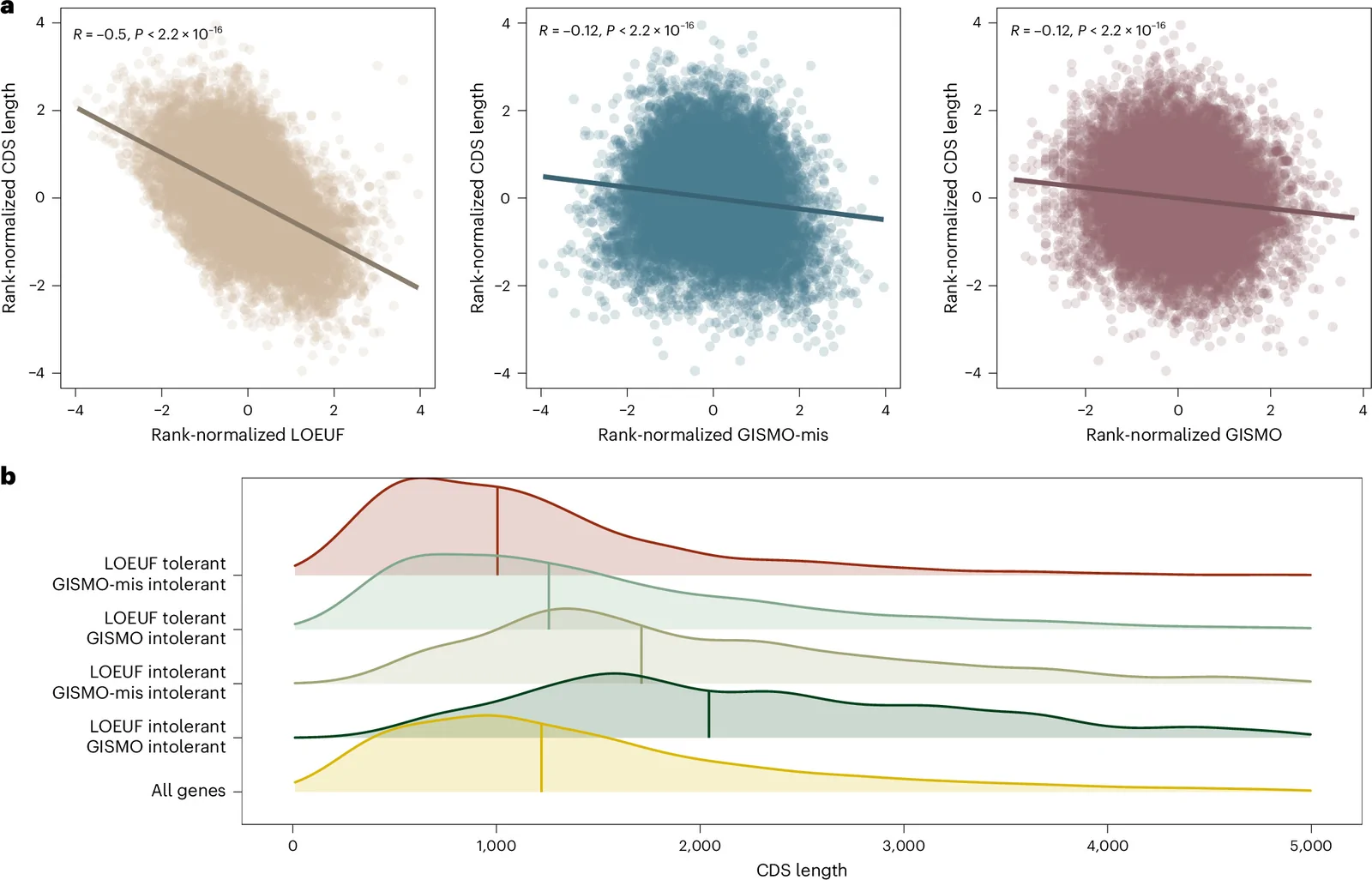
GISMO and GISMO-mis capture shorter genes relative to human constraint metrics. **a**, Correlation of GISMO, GISMO-mis and LOEUF against CDS length using a Spearman’s correlation test. The thicker colored line indicates the linear regression line across all data points. **b**, Short constrained GISMO and GISMO-mis genes considered tolerant for LOEUF. The colored line within the distributions indicates the median CDS length for that category.

Existing human constraint metrics are biased by gene length because short genes have smaller mutational targets and are often poorly calibrated. The correlation of GISMO and GISMO-mis with CDS length was *R* = −0.12, *P* = 3.8 × 10^−52^ for GISMO, *R* = −0.12, *P* = 4.0 × 10^−52^ for GISMO-mis and several magnitudes lower compared to LOEUF (*R* = −0.50, *P* < 1 × 10^−300^) (Fig. 3a). As LOEUF may be poorly calibrated for short genes, we tested whether GISMO and GISMO-mis identified shorter intolerant genes. For each metric, we defined intolerance as the top 15% most constrained genes. Both GISMO and GISMO-mis identified much shorter intolerant genes (GISMO median CDS = 1,502 bp for 2,897 genes and GISMO-mis median CDS = 1,302 bp for 2,502 genes) than LOEUF-intolerant genes (median CDS = 2,267 bp for 2,968 genes). We additionally found that genes predicted to be intolerant by GISMO and GISMO-mis but not LOEUF tend to be much shorter compared to genes that are intolerant to LOEUF and one of the GISMO metrics (Fig. 3b).

The genes that were considered tolerant for LOEUF (>0.35) and GISMO intolerant were enriched for genes involved in the following pathways or gene sets in mice: sperm immotility (Bonferroni’s adjusted *P* (*P*_adj_) = 2.17 × 10^−3^, 23 of 46 genes), absent acrosome (*P*_adj_ = 4.86 × 10^−2^, 16 of 30 genes) and abnormal actin cytoskeleton morphology (*P*_adj_ = 4.86 × 10^−2^, 15 of 27 genes) (Supplementary Table 5).

### Identifying potential sex chromosome biases in LOEUF

We also examined genes that appear highly constrained by LOEUF but fall into the least constrained GISMO deciles—that is, genes that are intolerant to heterozygous LoF in humans yet frequently lost across mammals. This discordant set was notably enriched for X-linked genes, raising the possibility that LOEUF may overestimate constraint for some sex-linked loci due to hemizygosity, sex-biased selection or technical challenges in variant detection from short-read sequencing (Supplementary Table 6). When restricting to X-linked genes only, we found no significant enrichment for Mendelian disease genes or essential phenotypes, suggesting that these discrepancies may reflect biological or technical factors specific to human population data rather than true evolutionary indispensability (Supplementary Table 7).

### Mammalian gene loss and fixed missense differences capture human disease signals

**Fig. 4:**
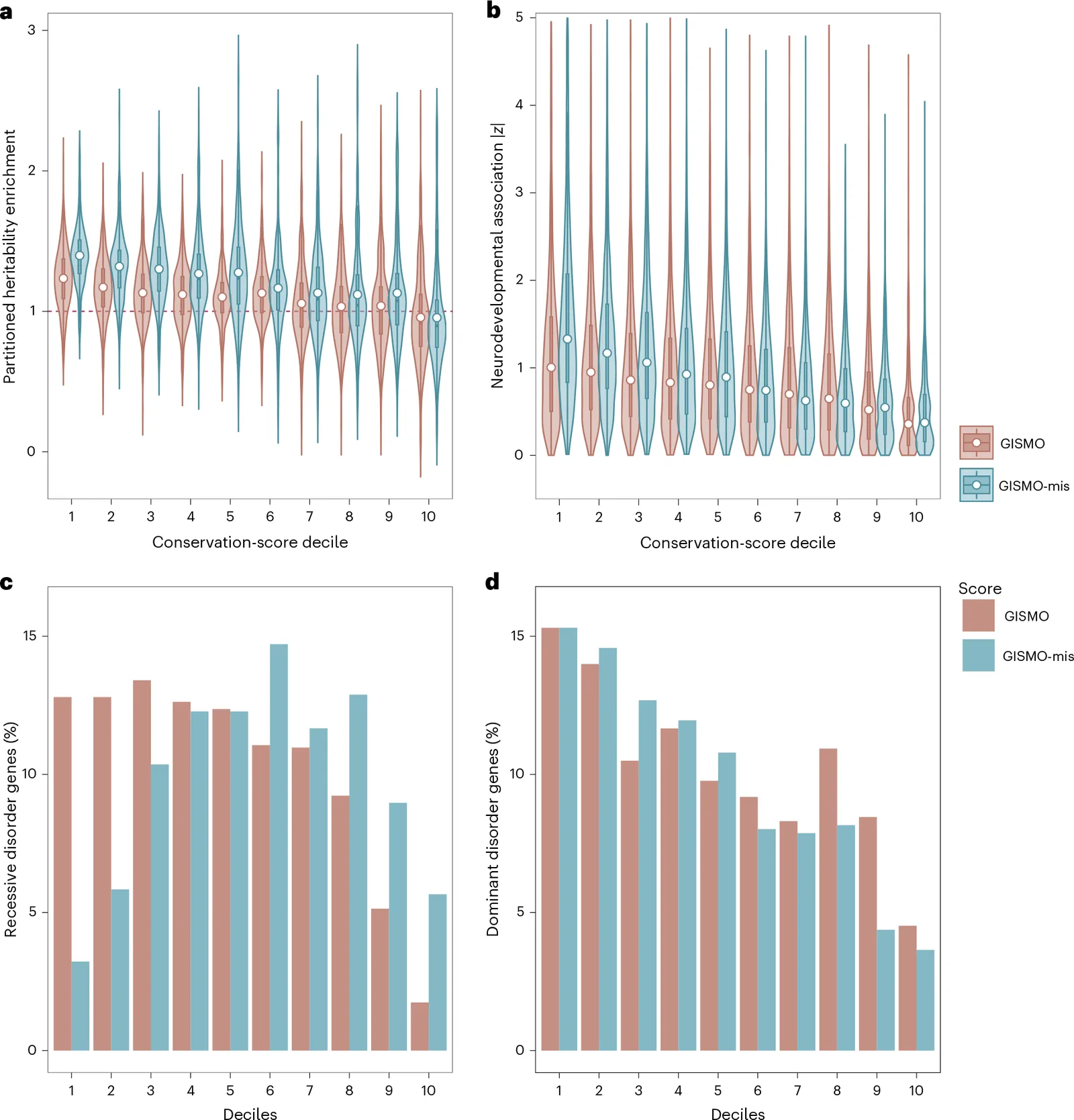
GISMO and GISMO-mis capture the disease signal and GISMO captures the recessive signal. **a**, Mean enrichment of per-SNV partitioned heritability of 274 traits explained by common variants within 100 kb of genes for GISMO and GISMO-mis deciles. The dashed horizontal line indicates the null expectation of 1. Violin plots show the distribution of values within each conservation-score decile. Embedded box plots indicate the median and IQR, with whiskers extending to 1.5× the IQR. The white circles indicate the mean. **b**, Mean neurodevelopmental disorder association across GISMO and GISMO-mis deciles. The association reflects *P* values estimated from the transmission and de novo association test of neurodevelopmental disorder rare variant associations that were later converted to absolute *z*-scores. Violin plots show the distribution of values within each conservation-score decile. Embedded box plots indicate the median and IQR with whiskers extending to 1.5× the IQR. The white circles indicate the mean. **c**, The percentage of genes present in each decile of GISMO and GISMO-mis for 1,183 recessive disorder genes aggregated from Online Mendelian Inheritance in Man (OMIM). **d**, The percentage of genes present in each decile of GISMO and GISMO-mis for 709 dominant disorder genes aggregated from OMIM.

We next tested whether mammalian gene loss and fixed missense differences prioritize human disease genes. As many disease models are mammalian, conservation may identify genes amenable to modeling in mammals. First, we assessed common variant heritability. For both metrics, we partitioned heritability across 276 independent traits from the UK Biobank and across several disease traits not well captured in the biobank. We found that there was a strong linear enrichment of heritability across the deciles of both metrics, with lowest enrichment in the least constrained deciles (Fig. 4a and Supplementary Table 8). The partitioned heritability results were consistent across flanking thresholds (10 kb, 50 kb and 100 kb). Second, we looked at rare variant association studies of traits with decreased fecundity, such as autism, schizophrenia and neurodevelopmental disorders. We found that both GISMO-constrained and GISMO-mis-constrained genes had a higher association with neurodevelopmental disorders and the metrics were strongly correlated with the association strength (Fig. 4b and Supplementary Figs. 6 and 7).

### Improving recessive disease–gene prioritization

Given that gene loss in a species can reflect selection on homozygous LoF carriers, we hypothesized that GISMO could be used to prioritize recessive genes associated with disease (that is, genes under homozygous selection should be more constrained by GISMO). To assess this, we assembled a list of 1,183 recessive genes and found that there was enrichment among the most constrained deciles (OR = 3.37 (95% CI 2.91–3.89), *P* = 1.42 × 10^−52^; Fig. 4c), whereas GISMO-mis expectedly did not have a significant enrichment (OR = 0.91 (0.73–1.13), *P* = 0.41). We additionally found that both GISMO and GISMO-mis prioritize enrichment in dominant disorder genes in the most constrained deciles (OR_GISMO_ = 9.92 (95% CI 8.07–12.20), *P* = 1.28 × 10^−94^; OR_GISMO-mis_ = 4.38 (3.67–5.22), *P* = 3.46 × 10^−52^) (Fig. 4d). Next, we sought to test whether GISMO may help with prioritization of recessive inheritance candidate genes in the Deciphering Developmental Disorders study consisting of 14,000 trios. We found that the GISMO distribution for recessive developmental disorder genes with a high confidence (confident or strong) followed similar distributions to Mendelian recessive genes, whereas the recessive genes with limited and moderate evidence were unclear (Supplementary Fig. 8). We also found that the limited and moderate evidence categories prioritized genes with high constraint for LOEUF, a reflection of strong heterozygous selection, which are unlikely gene candidates for recessive disorders. To further investigate divergence between cross-species and human-specific constraint, we examined known autosomal recessive disease genes from the curated set of definitive recessive genes in the Deciphering Developmental Disorders study, which fall into the least constrained GISMO deciles. Although this subset is small, enrichment analysis revealed phenotypic associations related to corneal development, thoracic structure and skeletal morphogenesis (Supplementary Table 7). These findings suggest that some genes may be essential in humans yet dispensable across mammals due to lineage-specific compensation, ecological specialization or relaxed evolutionary pressures.

### Recommended usages across mammalian and human genetics

GISMO and GISMO-mis are complementary gene-level conservation metrics derived from placental mammal alignments. GISMO quantifies gene-loss frequency across the phylogeny, whereas GISMO-mis measures the depletion of missense relative to synonymous substitutions. Both can be applied in human and comparative mammalian genomics.

We recommend that both GISMO and GISMO-mis be used as continuous variables whenever possible, to preserve the full information content across the spectrum of conservation. However, for applications requiring categorical classification, decile-based thresholds may be employed. Below we outline cases of recommended use and recommended strategies for interpretation.

In human genetics, the most constrained deciles (for example, deciles 1–2) are enriched for genes that are dosage sensitive, cause early lethality in model organisms or are associated with Mendelian disease. GISMO-mis, in particular, shows strong concordance with human constraint metrics such as LOEUF and AlphaMissense and is informative for polygenic traits when used continuously or in broader decile windows (for example, the top 20–40%). Notably, GISMO retains scoring power in duplicated or poorly mappable genes that are often excluded from short-read human constraint models.

In comparative mammalian genomics, GISMO enables the identification of genes that are broadly retained across mammals, reflecting long-term evolutionary indispensability. Conversely, high GISMO values may highlight lineage-specific dispensability or adaptive gene loss. GISMO-mis complements this by identifying codon-level amino acid conservation, which can inform studies of protein evolution, functional domain constraint or convergent adaptation.

Importantly, conservation-based metrics are agnostic to demographic features of contemporary human populations and can offer more reliable estimates in regions where human constraint models may be biased or underpowered. For instance, we observe that genes on the X chromosome are disproportionately assigned strong constraint scores by LOEUF, even when conservation metrics suggest that they are moderately or weakly constrained (Supplementary Table 9). This discrepancy likely reflects miscalibration of LOEUF in regions of reduced effective population size, skewed mutation rates or sex-specific selection—factors that complicate modeling of constraint from standing human variation. In contrast, GISMO and GISMO-mis show more uniform constraint distributions across autosomes and sex chromosomes, underscoring the value of incorporating deep-time conservation into gene prioritization frameworks.

These metrics may also facilitate phylogenetic profiling, pathway enrichment analyses and cross-species annotation transfer. To support application-specific thresholding, we recommend calibrating cutoffs empirically against known gene sets (for example, ClinGen annotations, mouse knockout phenotypes or disease–gene catalogs). A summary of recommended applications is provided in Table 1, but we emphasize that these are not the only cases.

**Table 1:** Recommended cases for use in GISMO and GISMO-mis

Application	Metric(s)	Usage	Suggested threshold	Rationale
Mendelian disease–gene prioritization	GISMO, GISMO-mis	Decile based or continuous	Deciles 1–2 (most constrained)	Genes under strong evolutionary constraint often overlap essential loci
Recessive disease–gene discovery	GISMO	Decile based	Deciles 1–4	GISMO enriches for known recessive disease genes
Pathway-level analyses	GISMO, GISMO-mis	Continuous	Not available (N/A)	Allows ranked enrichment tests or gene-set filtering
Cross-mammalian conservation	GISMO, GISMO-mis	Continuous	N/A	Captures gene or codon-level retention across 180 million years of evolution
Lineage-specific evolution	GISMO	Continuous	N/A	Identifies clade-specific gene loss, adaptation or duplication
Analysis of duplicated genes	GISMO	Continuous	N/A	GISMO retains scoring power where LOEUF or probability of LoF interference often fails due to mapping bias

## Discussion

Comparative genomics leverages millions of years of evolution to identify biologically important genes. Across placental mammals representing ~10% of recognized species, we characterized gene loss and fixed missense differences. Although most genes are retained across mammals, each species has >1,000 likely gene losses, concentrated among genes tolerant to disruption. Missense fixation events are similarly more frequent in less-constrained genes. We developed GISMO and GISMO-mis to quantify these complementary signals of selection.

GISMO is one of the first metrics to quantify gene loss across the mammalian tree. Although previous conservation-based methods have focused primarily on single-variant effects, GISMO expands on the well-established principle of evolutionary conservation by quantifying conservation at the gene level across deep evolutionary timescales^28^. We found that GISMO is well saturated, given that only six genes did not have any gene loss events. Among these six conserved genes, we found that they were related to DNA replication, poly(A) binding for RNA and development. This is in contrast to human-based metrics from gnomAD such as LOEUF and probability of LoF intolerance (pLI), where >1,000 genes do not have an observed LoF event across 141,456 individuals in gnomAD v2.1 and 832 genes across 807,162 individuals in gnomAD v4.0. More importantly, we found that only 2,483 genes have at least 1 homozygous LoF carrier in gnomAD v2.1, whereas v4.0 is currently unreported. Given that gene loss is determined by disruptive open reading frames in a genome assembly, this suggests that these gene loss events are fixed. This may be seen analogously as quantifying homozygous LoF carriers in intraspecies analyses, which may explain why GISMO can prioritize clinical recessive disease genes.

We found that most genes that were likely to be human specific tended to be enriched for single exons and pseudogenes, likely reflecting potential noise or events that are not real or biologically meaningful. We benchmarked these metrics against known gene sets and model organism data and highlighted the potential for identifying disease genes and function.

GISMO and GISMO-mis identify short, conserved genes under selection that may be missed by LOEUF. As gene essentiality is not determined by gene length, length-robust metrics improve characterization of the functional spectrum. GISMO also prioritizes recessive disease genes, which most constraint metrics are not calibrated to identify. Prior studies have highlighted how recessive metrics such as pRec, which measures the probability of a gene being recessive, cannot differentiate weak selection on heterozygotes from that on homozygotes, a limitation of human genetic data^29^. We further showed that combining GISMO and heterozygous selection metrics such as LOEUF can help improve clinical prioritization of genes. For instance, a gene candidate is much more likely to be recessive if the GISMO score is low and the LOEUF score high. We additionally found that GISMO-intolerant and LOEUF-tolerant genes are important for fertility and reproductive success, with pathway enrichments such as immotile sperm in mice.

Notably, we found that genes with high LOEUF constraint but low GISMO conservation were enriched on the X chromosome, suggesting that LOEUF may overestimate constraint for certain sex-linked genes due to hemizygosity or technical artifacts. These genes did not show enrichment for essential or Mendelian phenotypes when restricted to the X chromosome, indicating that their discordant scores may reflect human-specific or sex-biased pressures rather than broad evolutionary constraint. We further emphasized that the strong correlations between GISMO and human constraint metrics highlight the power of interspecies over intraspecies metrics, in particular, the high costs and sample sizes required to generate calibrated intraspecies human constraint metrics such as LOEUF (>800,000 individuals) and pHaplo or pTriplo (>1 million individuals) in contrast to 462 mammals for GISMO.

Moreover, in human genetics, constraint metrics have been transformative for many different subfields. In particular, the pLI and subsequent LOEUF metrics, derived from large-scale sequencing studies, have become standard parts of genetic and genomic analysis pipelines. These metrics have revolutionized a number of workflows in human genetics, including filtering and prioritization of genes and how likely they are to have phenotypic impact for association studies and identification of which genes or variants are likely under heterozygous LoF selection, as well as clinical interpretation of patient variation. Importantly, the GISMO metrics will allow nonhuman mammalian researchers to have metrics to further enable and advance the analytical framework in the large field.

Similarly, GISMO-mis builds on the classic dN-to-dS ratio measure, which quantifies the ratio of nonsynonymous (missense) to synonymous substitutions to infer selective pressure^30–32^. However, GISMO-mis innovatively leverages a multi-species consensus approach, incorporating evolutionary distances from humans and employing robust methods for handling ambiguous alignments. This provides a scalable, reliable and biologically relevant metric that complements traditional dN-to-dS ratio measures, offering enhanced correlation with human-specific constraint metrics such as LOEUF and AlphaMissense, as well as improved identification of clinically relevant genes.

An open question from these analyses was whether mammalian gene loss and fixed missense differences could be leveraged to capture important biological insights into human diseases and traits. To explore this question, we analyzed an array of quantitative traits, human disease phenotypes and gene sets robustly associated with rare and common diseases. We found significant common variant heritability enrichment across a vast number of traits in the most constrained deciles of both GISMO and GISMO-mis. We similarly found that both metrics capture rare variant association to traits with decreased fecundity. We posit that GISMO and GISMO-mis can provide orthogonal levels of disease evidence and may help with increasing disease association power. Moreover, we show that most of the rare and common variant heritability is concentrated in genes that are strongly conserved across species. This reinforces the potential utility of mammalian models for dissection of heritable quantitative traits in humans.

Despite the increased power and clear value demonstrated herein for derived metrics based on mammalian orthologs such as GISMO and GISMO-mis, there remain several limitations in the derivation of these metrics. Mammalian gene conservation may not reflect the human selective pressure and recent innovation that has affected the selection regimen of certain traits. It is also important to understand the practical use of GISMO given that it taps into both homozygous and heterozygous selection; pairing with additional constraint metrics such as LOEUF and GISMO-mis can help tease apart these relationships. Moreover, the species included may be subjected to survivorship bias and accessibility bias, so further sampling of a broader spectrum of mammals will improve metrics such as GISMO and GISMO-mis. Furthermore, despite its nonphylogenetic nature—meaning that it does not differentiate between multiple independent loss events and a single ancestral event—GISMO consistently outperforms a phylogenetically adjusted alternative when correlated with independent measures of functional constraint. This superior performance likely arises from the stringent criteria imposed by TOGA, which requires multiple disruptions in the open reading frame to classify a gene as lost. In effect, only those loss events supported by several independent mutation occurrences contribute to the GISMO score, thereby mitigating the risk of overcounting and robustly capturing the essential signal of gene conservation. Nevertheless, for researchers who prefer an approach that explicitly accounts for evolutionary relationships, we have also released a phylogenetic version of GISMO. Finally, recent human specific duplication events may show a slight correlation with the conservation score of the original paralog; however, these are few and largely a limitation across all constraint and conservation metrics that currently exist.

Overall, we demonstrate that mammalian gene loss and missense fixation are important measures of selection. We developed several powerful metrics to quantify evolutionary constraints for gene loss (GISMO) and molecular adaptation (GISMO-mis). Our estimates provide informative ranking of gene importance, which ultimately allow us to better understand gene essentiality and disease association.

## Methods

### Development of the GISMO metric

To infer orthologs, we used TOGA^25^ with the human GENCODE 38 annotation^33^ across 462 mammals. As, for some species, TOGA data were available for multiple assemblies, we selected only the assembly with the highest contig N50. An orthologous gene is considered lost when and only when all transcripts of the respective gene are categorized as lost. To assess the type of orthology, TOGA subsequently evaluates, for each human reference gene, the classification of all its corresponding orthologous loci and which reference genes were annotated. Gene loss is defined as disruption of the open reading frame in the transcript, with more disruptions indicating higher confidence in gene loss. These gene loss events are synonymously referred to as one2zero events. A 95% CI was simulated using a binomial distribution and the upper 95% CI was used. Briefly, the binomial probability was estimated for each phylogenetic order and counts were simulated 10,000× for the 462 species. Each count was subsequently weighted by the evolutionary distance relative to humans, where 1 = gene loss. Evolutionary distance was standardized by dividing by the maximum evolutionary distance among the mammals used to generate GISMO, represented by ${\hat{p}}_{i}$ below.

For gene *i* and species *j* define:

$${\hat{p}}_{i}=\frac{\mathop{\sum }\nolimits_{j=1}^{462}{I}_{{ij}}{w}_{\!j}}{\mathop{\sum }\nolimits_{j=1}^{462}{w}_{\!j}}$$

where:

$${I}_{i,\;j}=\left\{\begin{array}{ll}1,\qquad{\mathrm{if\,gene}}\,i\,{\mathrm{is\,lost\,in\,species\,relative\,to\,humans}}\\ 0,\qquad{\mathrm{otherwise}}\end{array}.\right.$$

Let *d*_*j*_ be the evolutionary distance (in millions of years) between humans and species *j*, obtained from the TimeTree database, and *D*_max_ is defined as:

$${D}_{\max }={\max }_{j=1}^{462}{d}_{j}.$$

We assigned a weight:

$${w}_{\!j}=\frac{{d}_{\!j}}{{D}_{\max }}$$

so that *w*_*j*_ ranges between 0 and 1. Losses in species more distantly related to humans contribute a larger weight. A smaller GISMO value indicates that a gene is rarely lost across mammals (that is, it is more constrained), because most *I*_*ij*_ values will be zero. Although losses in more-distant species contribute more weight, this is intentional: if a gene is maintained in species that diverged hundreds of millions of years ago, it strongly suggests essential function over deep evolutionary time.

To estimate a CI on $\hat{p}$, we simulated data for *I*_*ij*_.

Let:

$${\widetilde{I}}_{{ijk}} \sim {{\mathrm{Bernoulli}}}\left({\hat{p}}_{i}\right)$$

$${{\mathrm{for}}}\,k\,{{\mathrm{in}}}\{1\ldots 10,000\}.$$

We can calculate the set of 10,000:

$${\widetilde{p}}_{{ik}}=\frac{\mathop{\sum }\nolimits_{j=1}^{462}{I}_{{ijk}}{w}_{j}}{\mathop{\sum }\nolimits_{j=1}^{462}{w}_{j}}$$

and find the 95th percentile of ${\widetilde{p}}_{{ik}}$ as an estimate of ${\hat{p}}_{i}$.

### Development of GISMO-mis

To calculate GISMO-mis, multiple codon alignments, including up to 462 mammals, have been generated using MACSE (v2)^34^ and were downloaded from http://genome.senckenberg.de/download/TOGA/. To generate codon alignments, the following selection procedures were performed: (1) human transcript with the longest coding sequence length; and (2) orthologs considered if they were classified as intact, partial intact or uncertain loss. To ensure alignments were mostly one-to-one orthologs, if a query species has more than four predicted orthologs, this species was not included in the multiple codon alignment. In addition, if the gene did not have a single ortholog for at least 75% of all query species, a multiple codon alignment was not computed for this gene. Subsequently, for each gene, a mammalian consensus transcript sequence across all species was generated and used as a reference. To determine each consensus transcript sequence, all mammalian sequences for that gene were considered at a codon-level resolution and the most represented codon(s) across all queried species was chosen. In the event of tied codon counts, the consensus sequence permits multiple possible reference codons. Next, missense and synonymous counts across each species were quantified against the mammalian consensus sequence for each transcript. Any codon comparisons involving gaps in either reference or queried codons were not considered. In the case of multiple reference codons at a given position, missense and synonymous counts were conservatively generated by classifying a queried codon as missense only if none of the reference codons was synonymous with the queried codon. Similarly, in the case of a codon with ambiguous nucleotides, each ambiguous nucleotide was exhaustively replaced until either (1) a synonymous result was achieved, after which the query would be classified as synonymous, or (2) no synonymous result was achieved across all potential substitutions, after which the query would be classified as missense. Finally, for each gene, the mean missense to synonymous ratio across all queried species was calculated.

For gene *i* and species *j* define:

$${\hat{p}}_{i}=\mathop{\sum }\limits_{j=1}^{462}\frac{{m}_{{ij}}}{{s}_{{ij}}}$$

where *m*_*ij*_ represents missense counts relative to the mammalian consensus sequence and *s*_*ij*_ represents the synonymous counts relative to the mammalian consensus sequence.

### Benchmarking against gene sets and pathways

To benchmark GISMO, we compared GISMO against several independent gene sets: lethal mouse, olfactory, essential genes and nonessential genes. These were the same gene sets previously used in gnomAD benchmarking that have a reasonable comparison^2^. Both GISMO and GISMO-mis were split into deciles, where the lowest decile (first) represented the most constrained. In addition, data from GTEx was used to assess how expressed genes are across the different number of tissues. Genes were considered expressed in a tissue with transcripts per million >0.3. Pathway enrichment was done using https://toppgene.cchmc.org/. A recessive gene set was curated from OMIM^35^, which included a total of 1,183 autosomal recessive genes.

### Correlation between GISMO and GISMO-mis against independent constraint metrics

To assess whether GISMO and GISMO-mis are associated with different constraint scores, both metrics were correlated against other gene-level metrics. First, the GISMO metrics were compared against two human constraint metrics derived from gnomAD for LoF constraint (LOEUF) and missense constraint (MOEUF). Next, to test whether GISMO and GISMO-mis can capture CNV dosage sensitivity, the scores were benchmarked against pTriplo and pHaplo^27^. In addition, we benchmarked GISMO and GISMO-mis against gene-level AlphaMissense scores^36^, which measure the effects of missense variation on predicted structural context from AlphaFold. A Spearman’s correlation was used for all correlations.

### Partitioned heritability across independent traits

To assess the distribution of heritability enrichment across GISMO and GISMO-mis, we used linkage disequilibrium (LD) score regression to partition heritability across gene deciles of both metrics^37–39^. For both metrics, we included a 100-kb flanking region both upstream and downstream for each gene and in conjunction with genotype data from the 1000 Genomes Project. The SNPs were restricted to HapMap3 SNPs with an estimated annotation-specific LD score using a 1-cM window. Next, partitioned heritability was applied to 276 independent traits from the UK Biobank (https://www.nealelab.is/uk-biobank/), as well as additional disease traits such as schizophrenia, bipolar disorder, autism spectrum disorder, attention-deficit hyperactivity disorder and coronary artery disease^40–44^. Phenotypes were selected from the UK Biobank based on having a significant *P* value (*P* < 0.05) after Bonferroni’s correction. In addition, phenotypes were assessed for phenotypic correlation and only independent traits were included. The baseline model (v2.2), which includes 74 annotations to capture genomic properties, was included alongside the estimated LD scores as a predictor in the LD score regression. HapMap3 SNPs, excluding the human leukocyte antigen region, were used as default regression SNPs.

### Assessing GISMO and GISMO-mis CDS biases

To assess whether GISMO and GISMO-mis are biased by CDS length, we correlated against the CDS length of each gene in both metrics. We compared against the gold standard for LoF constraint, LOEUF from gnomAD. Given that LOEUF had a significantly stronger correlation with CDS length relative to GISMO and GISMO-mis, we hypothesized that the GISMO metrics may help prioritize short essential genes. We considered genes with a LOEUF score <0.35 as LOEUF constrained and intolerant, representing roughly the top 15% most constrained genes. Similarly, for both GISMO and GISMO-mis, we took the top 15% most constrained genes, which we considered intolerant. We compared the CDS length distribution for GISMO and GISMO-mis intolerant and LOEUF tolerant.

### Reporting summary

Further information on research design is available in the Nature Portfolio Reporting Summary linked to this article.

## Online content

Any methods, additional references, Nature Portfolio reporting summaries, source data, extended data, supplementary information, acknowledgements, peer review information; details of author contributions and competing interests; and statements of data and code availability are available at https://doi.org/10.1038/s41588-026-02783-9.

## Supplementary information

Supplementary Information Supplementary Note and Figs. 1–8.

Reporting Summary

Supplementary Tables Supplementary Tables 1–11.

## Data Availability

All relevant data are available via Zenodo at https://doi.org/10.5281/zenodo.20547722 (ref. ^45^) and in Supplementary Tables 1–11.

## Code availability

All relevant code data are available via Zenodo at https://doi.org/10.5281/zenodo.20547722 (ref. ^45^).

## References

[1] Lek, M. et al. Analysis of protein-coding genetic variation in 60,706 humans. *Nature* **536**, 285–291 (2016). 10.1038/nature19057 PMC5018207 27535533

[2] Karczewski, K. J. et al. The mutational constraint spectrum quantified from variation in 141,456 humans. *Nature* **581**, 434–443 (2020). 10.1038/s41586-020-2308-7 PMC7334197 32461654

[3] Kaplanis, J. et al. Evidence for 28 genetic disorders discovered by combining healthcare and research data. *Nature* **586**, 757–762 (2020). 10.1038/s41586-020-2832-5 PMC7116826 33057194

[4] Agarwal, I., Fuller, Z. L., Myers, S. R. & Przeworski, M. Relating pathogenic LoF mutations in humans to their evolutionary fitness costs. *eLife* **12**, e83172 (2023). 10.7554/eLife.83172 PMC9937649 36648429

[5] Zuk, O. et al. Searching for missing heritability: designing rare variant association studies. *Proc. Natl Acad. Sci. USA* **111**, E455–E464 (2014). 10.1073/pnas.1322563111 PMC3910587 24443550

[6] Walker, L. C. et al. Using the ACMG/AMP framework to capture evidence related to predicted and observed impact on splicing: recommendations from the ClinGen SVI Splicing Subgroup. *Am. J. Hum. Genet.* **110**, 1046–1067 (2023). 10.1016/j.ajhg.2023.06.002 PMC10357475 37352859

[7] Gudmundsson, S. et al. Variant interpretation using population databases: lessons from gnomAD. *Hum. Mutat.* **43**, 1012–1030 (2022). 10.1002/humu.24309 PMC9160216 34859531

[8] Fu, J. M. et al. Rare coding variation provides insight into the genetic architecture and phenotypic context of autism. *Nat. Genet.* **54**, 1320–1331 (2022). 10.1038/s41588-022-01104-0 PMC9653013 35982160

[9] Singh, T. et al. Rare coding variants in ten genes confer substantial risk for schizophrenia. *Nature* **604**, 509–516 (2022). 10.1038/s41586-022-04556-w PMC9805802 35396579

[10] Palmer, D. S. et al. Exome sequencing in bipolar disorder identifies *AKAP11* as a risk gene shared with schizophrenia. *Nat. Genet.* **54**, 541–547 (2022). 10.1038/s41588-022-01034-x PMC9117467 35410376

[11] Epi25 Collaborative. Exome sequencing of 20,979 individuals with epilepsy reveals shared and distinct ultra-rare genetic risk across disorder subtype. *Nat. Neurosci.* **27**, 1864–1979 (2024). 10.1038/s41593-024-01747-8 PMC11646479 39363051

[12] Minikel, E. V. et al. Evaluating drug targets through human loss-of-function genetic variation. *Nature* **581**, 459–464 (2021). 10.1038/s41586-020-2267-z PMC7272226 32461653

[13] Ng, P. C. & Henikoff, S. SIFT: predicting amino acid changes that affect protein function. *Nucleic Acids Res.* **31**, 3812–3814 (2003). 10.1093/nar/gkg509 PMC168916 12824425

[14] Rentzsch, P., Witten, D., Cooper, G. M., Shendure, J. & Kircher, M. CADD: predicting the deleteriousness of variants throughout the human genome. *Nucleic Acids Res.* **47**, D886–D894 (2019). 10.1093/nar/gky1016 PMC6323892 30371827

[15] Ioannidis, N. M. et al. REVEL: an ensemble method for predicting the pathogenicity of rare missense variants. *Am. J. Hum. Genet.* **99**, 877–885 (2016). 10.1016/j.ajhg.2016.08.016 PMC5065685 27666373

[16] Pejaver, V. et al. Inferring the molecular and phenotypic impact of amino acid variants with MutPred2. *Nat. Commun.* **11**, 5918 (2020). 10.1038/s41467-020-19669-x PMC7680112 33219223

[17] Pancotti, C. et al. Predicting protein stability changes upon single-point mutation: a thorough comparison of the available tools on a new dataset. *Brief Bioinform.* **23**, bbab555 (2022). 10.1093/bib/bbab555 PMC8921618 35021190

[18] Koonin, E. V. Orthologs, paralogs, and evolutionary genomics. *Annu. Rev. Genet.* **39**, 309–338 (2005). 10.1146/annurev.genet.39.073003.114725 16285863

[19] Austin, C. P. et al. The knockout mouse project. *Nat. Genet.* **36**, 921–924 (2004). 10.1038/ng0904-921 PMC2716027 15340423

[20] Susaki, E. A., Ukai, H. & Ueda, H. R. Next-generation mammalian genetics toward organism-level systems biology. *npj Syst. Biol. Applic.* **3**, 15 (2017). 10.1038/s41540-017-0015-2 PMC5459797 28649442

[21] Jones, K. E. & Safi, K. Ecology and evolution of mammalian biodiversity. *Philos. Trans. R. Soc. Lond. B* **366**, 2451–2461 (2011). 10.1098/rstb.2011.0090 PMC3138616 21807728

[22] Christmas, M. J. et al. Evolutionary constraint and innovation across hundreds of placental mammals. *Science* **380**, eabn3943 (2023). 10.1126/science.abn3943 PMC10250106 37104599

[23] Nehrt, N. L., Clark, W. T., Radivojac, P. & Hahn, M. W. Testing the ortholog conjecture with comparative functional genomic data from mammals. *PLoS Comput. Biol.* **7**, e1002073 (2011). 10.1371/journal.pcbi.1002073 PMC3111532 21695233

[24] Peterson, M. E. et al. Evolutionary constraints on structural similarity in orthologs and paralogs. *Protein Sci.* **18**, 1306–1315 (2009). 10.1002/pro.143 PMC2774440 19472362

[25] Kirilenko, B. M. et al. Integrating gene annotation with orthology inference at scale. *Science* **380**, eabn3107 (2023). 10.1126/science.abn3107 PMC10193443 37104600

[26] Babadi, M. et al. GATK-gCNV enables the discovery of rare copy number variants from exome sequencing data. *Nat. Genet.* **55**, 1589–1597 (2023). 10.1038/s41588-023-01449-0 PMC10904014 37604963

[27] Collins, R. L. et al. A cross-disorder dosage sensitivity map of the human genome. *Cell* **185**, 3041–3055 (2022). 10.1016/j.cell.2022.06.036 PMC9742861 35917817

[28] Capriotti, E. & Fariselli, P. Evaluating the relevance of sequence conservation in the prediction of pathogenic missense variants. *Hum. Genet.* **141**, 1649–1658 (2022). 10.1007/s00439-021-02419-4 35098354

[29] Fuller, Z. L., Berg, J. J., Mostafavi, H., Sella, G. & Przeworski, M. Measuring intolerance to mutation in human genetics. *Nat. Genet.* **51**, 772–776 (2019). 10.1038/s41588-019-0383-1 PMC6615471 30962618

[30] Li, W. H. Unbiased estimation of the rates of synonymous and nonsynonymous substitution. *J. Mol. Evol.* **36**, 96–99 (1993). 10.1007/BF02407308 8433381

[31] Goldman, N. & Yang, Z. A codon-based model of nucleotide substitution for protein-coding DNA sequences. *Mol. Biol. Evol.* **11**, 725–736 (1994). 10.1093/oxfordjournals.molbev.a040153 7968486

[32] Nielsen, R. & Yang, Z. Likelihood models for detecting positively selected amino acid sites and applications to the HIV-1 envelope gene. *Genetics* **148**, 929–936 (1998). 10.1093/genetics/148.3.929 PMC1460041 9539414

[33] Frankish, A. et al. GENCODE 2021. *Nucleic Acids Res.* **49**, D916–D923 (2021). 10.1093/nar/gkaa1087 PMC7778937 33270111

[34] Ranwez, V., Douzery, E. J. P., Cambon, C., Chantret, N. & Delsuc, F. MACSE v2: toolkit for the alignment of coding sequences accounting for frameshifts and stop codons. *Mol. Biol. Evol.* **35**, 2582–2584 (2018). 10.1093/molbev/msy159 PMC6188553 30165589

[35] Hamosh, A., Scott, A. F., Amberger, J. S., Bocchini, C. A. & McKusick, V. A. Online Mendelian Inheritance in Man (OMIM), a knowledgebase of human genes and genetic disorders. *Nucleic Acids Res.* **33**, D514–D517 (2005). 10.1093/nar/gki033 PMC539987 15608251

[36] Cheng, J. et al. Accurate proteome-wide missense variant effect prediction with AlphaMissense. *Science* **381**, eadg7492 (2023). 10.1126/science.adg7492 37733863

[37] Finucane, H. K. et al. Partitioning heritability by functional annotation using genome-wide association summary statistics. *Nat. Genet.* **47**, 1228–1235 (2015). 10.1038/ng.3404 PMC4626285 26414678

[38] Finucane, H. K. et al. Heritability enrichment of specifically expressed genes identifies disease-relevant tissues and cell types. *Nat. Genet.* **50**, 621–629 (2018). 10.1038/s41588-018-0081-4 PMC5896795 29632380

[39] Bulik-Sullivan, B. K. et al. LD score regression distinguishes confounding from polygenicity in genome-wide association studies. *Nat. Genet.* **47**, 291–295 (2015). 10.1038/ng.3211 PMC4495769 25642630

[40] Grove, J. et al. Identification of common genetic risk variants for autism spectrum disorder. *Nat. Genet.* **51**, 431–444 (2019). 10.1038/s41588-019-0344-8 PMC6454898 30804558

[41] Mullins, N. et al. Genome-wide association study of more than 40,000 bipolar disorder cases provides new insights into the underlying biology. *Nat. Genet.* **53**, 817–829 (2021). 10.1038/s41588-021-00857-4 PMC8192451 34002096

[42] Demontis, D. et al. Genome-wide analyses of ADHD identify 27 risk loci, refine the genetic architecture and implicate several cognitive domains. *Nat. Genet.* **55**, 198–208 (2023). 10.1038/s41588-022-01285-8 PMC10914347 36702997

[43] Tcheandjieu, C. et al. Large-scale genome-wide association study of coronary artery disease in genetically diverse populations. *Nat. Med.* **28**, 1679–1692 (2022). 10.1038/s41591-022-01891-3 PMC9419655 35915156

[44] Trubetskoy, V. et al. Mapping genomic loci implicates genes and synaptic biology in schizophrenia. *Nature* **604**, 502–508 (2022). 10.1038/s41586-022-04434-5 PMC9392466 35396580

[45] Liao, C. & R. Ye. cliao5/GISMO: GISMO v1.0.0. *Zenodo*10.5281/zenodo.20547722 (2026).

