# Supplementary material for: The landscape of gene loss and missense variation across the mammalian tree informs on gene essentiality
Source: Nat Genet. 2026 Oct 20;58(11):3037–45. doi: 10.1038/s41588-026-02783-9 (PMC13754592; doi:10.1038/s41588-026-02783-9)
Supplement: Supplementary file 1 [file 41588_2026_2783_MOESM1_ESM.pdf]

nature genetics

Supplementary information https://doi.org/10.1038/s41588-026-02783-9

# The landscape of gene loss and missense variation across the mammalian tree informs on gene essentiality

In the format provided by the authors and unedited

# Supplementary Text and Figures

## Development of the GISMO metric

First, we had to infer orthologs across existing mammalian assemblies. A large portion of new mammalian assemblies were derived from Zoonomia. To infer orthologs, the Tool to infer Orthologs from Genome Alignments (TOGA)(*20*) was used with the human GENCODE 38 annotation(*24*) across 462 mammals. Since for some species TOGA data was available for multiple assemblies, we selected only the assembly with the highest contig N50. An orthologous gene is considered lost when and only when all transcripts of the respective gene are categorized as lost. To assess the type of orthology, TOGA subsequently evaluates, for each human reference gene, the classification of all its corresponding orthologous loci and which reference genes were annotated. Ortholog classification results across 462 mammals (https://genome.senckenberg.de/download/TOGA/human_hg38_reference/), were used to construct a matrix cataloging every gene from every mammalian species as having a one-to-one, one-to-many, one-to-zero, many-to-one, or many-to-many ortholog correspondence.

A 95% confidence interval was simulated using a binomial distribution and the upper 95% confidence interval was used. Briefly, the binomial probability was estimated for each phylogenetic order and counts were simulated 10,000 times for the 462 species. We chose to use the upper confidence bound to make each gene score more conservative. Each count was subsequently weighted by the evolutionary distance relative to humans, where 1 = gene

loss. Evolutionary distance relative to humans was standardized by dividing by the maximum evolutionary distance amongst the mammals used to generate GISMO. We compared the metric with and without evolutionary distance weighting. We decided to move forward with the evolutionary distance weighting because there were marginal increases in correlation with human constraint metrics.

## Development of GISMO-mis

To calculate GISMO-mis, multiple codon alignments including up to 462 mammals have been generated using MACSE v2(25) and were downloaded from http://genome.senckenberg.de/download/TOGA/. To generate codon alignments, the following selection procedures were performed: 1) Human transcript with the longest coding sequence length, 2) Orthologs were considered if they were classified as intact, partial intact, or uncertain loss. To ensure alignments were mostly 1 to 1 orthologs, if a query species has more than four predicted orthologs, this species was not included in the multiple codon alignment. Additionally, if the gene did not have a single ortholog for at least 75% of all query species, a multiple codon alignment was not computed for this gene. Subsequently for each gene, a mammalian consensus transcript sequence across all species was generated and used as a reference. To determine each consensus transcript sequence, all mammalian sequences for that gene were considered at a codon-level resolution and the most represented codon(s) across all queried species was chosen. In the event of tied codon counts, the consensus sequence permits multiple possible reference

codons. Next, missense and synonymous counts across each species were quantified against the mammalian consensus sequence for each transcript. Any codon comparisons involving gaps in either reference or queried codons were not considered. In the case of multiple reference codons at a given position, missense and synonymous counts were conservatively generated by only classifying a queried codon as missense if none of the reference codons were synonymous with the queried codon. Similarly in the case of a codon with ambiguous nucleotides, each ambiguous nucleotide was exhaustively replaced until either 1) a synonymous result was achieved, after which the query would be classified as synonymous, or 2) no synonymous result was achieved across all potential substitutions, after which the query would be classified as missense. Finally for each gene, the mean missense to synonymous ratio across all queried species was calculated.

For gene $i$ and species define:

$$\hat{p}_i = \sum_{j=1}^{462} \frac{m_{ij}}{s_{ij}}$$

Where $m_{ij}$ represents missense counts relative to the mammalian consensus sequence and $s_{ij}$ represents the synonymous counts relative to the mammalian consensus sequence.

We additionally assessed whether weighing the missense and synonymous ratios by amino acid replacement scores would improve the association and benchmarking against human constraint. We assessed popular amino acid replacement scores such as Sneath's

index, Grantham's distance, and Miyata's distance. The amino acid replacement scores were standardized to 0 and 1. We found the weighting did not have any significant effect on capturing human constraint.

### Phylogenetic detection of gene loss events

We developed a phylogeny-aware approach to identify and quantify gene losses across mammals. Let $G = \{g_1, g_2, \ldots, g_n\}$ be the set of genes and $S = \{s_1, s_2, \ldots, s_m\}$ be the set of species. For each gene $g \in G$, we defined a binary presence/absence vector $X\_g = (x_1, x_2, \ldots, x_m)$, where $x_i = 1$ indicates gene presence and $x_i = 0$ indicates gene loss. These binary states were mapped onto a rooted mammalian phylogenetic tree $T = (V,E)$, where $V$ is the set of vertices (nodes) and $E$ is the set of edges (branches).

Using a bottom-up parsimony algorithm, we reconstructed ancestral states at internal nodes $V\_int \subset V$ by applying a majority rule to descendant nodes. For each internal node $v \in V\_int$ with descendant set $D(v)$, the ancestral state was computed as:

State($v$) = 1 if Σ_{d∈D(v)} State($d$) / |$D(v)$| ≥ 0.5 = 0 otherwise

Independent loss events were identified as transitions from presence (1) to absence (0) along tree branches. For each gene $g$, we defined the set of loss events $L\_g = \{l_1, l_2, \ldots, l_k\}$, where each loss event $l_i$ occurs on an edge $e \in E$ where Parent($e$) has state 1 and Child($e$) has state 0.

**Evolutionary Time-Weighted Scoring System**

We implemented two complementary weighting schemes based on species divergence times from humans. Let $D: S \rightarrow \mathbb{R}^+$ be a function that maps each species to its divergence time from humans in millions of years ago (MYA), obtained from the TimeTree database.

1. Ancient Loss Weighting: The ancient loss weighted score emphasizes losses in evolutionarily distant species:

$$\text{WLS\_ancient}(g) = \Sigma_{i=1}^{k} (l_i \times D(s_i))$$

where $s_i$ is the species associated with loss event $l_i$. For example, a loss in platypus (180 MYA) contributes more than a loss in mouse (75 MYA).

2. Recent Loss Weighting: The recent loss weighted score emphasizes losses in evolutionarily closer species:

$$\text{WLS\_recent}(g) = \Sigma_{i=1}^{k} (l_i \times (180 - D(s_i)))$$

where 180 MYA represents the maximum divergence time in our dataset.

For each gene, we calculated normalized scores to account for sampling density:

$$\text{WLS\_norm}(g) = \text{WLS}(g) / (|L\_g| \times \max(D))$$

**Comparison with Non-Phylogenetic Methods**

We compared our phylogenetic approach with GISMO, which identifies losses based solely on presence/absence patterns weighted by gene loss.

For statistical comparison between methods, we calculated Spearman's rank correlation coefficient $\rho$.

**Quality Control and Validation**

We implemented multiple validation steps to ensure data integrity:

1. Matrix validation: $\forall g \in G$, $\forall s \in S$: $x_{g,s} \in \{0,1\}$
2. Tree completeness: $|V_{tip}| = |S|$
3. Divergence time consistency: $\forall s \in S$: $0 \leq D(s) \leq 180$

Edge cases were specifically tested, including genes with:

- Complete presence ($\forall s \in S$: $x_{g,s} = 1$)
- Complete absence ($\forall s \in S$: $x_{g,s} = 0$)
- Single loss events
- Multiple independent losses

All analyses were performed using R (version 4.1.0) with the ape package for phylogenetic operations. Statistical analyses and visualizations were conducted using the tidyverse suite of packages. The complete analysis pipeline, including data preprocessing, loss detection, score calculation, and comparative analysis, is available on GitHub.

### Benchmarking against gene sets and pathways

To test the robustness of our new scores, we sought to identify independent datasets to determine whether the scores sufficiently capture these independent metrics and expected gene

sets. To benchmark GISMO, we compared GISMO against several independent gene sets: lethal mouse, olfactory, essential genes, and non-essential genes. These were the same genesets previously used in gnomAD benchmarking to have a reasonable comparison(2). Both GISMO and GISMO-mis were split into deciles, where the lowest decile (1st) represented the most constrained. Additionally, data from GTEx 53 was used to assess how expressed genes are across the different number of tissues. Genes were considered expressed in a tissue with a TPM >0.3. Pathway enrichment was done using https://toppgene.cchmc.org/. A recessive gene set was curated from OMIM(26), which included a total of 1,183 autosomal recessive genes.

### Correlation between GISMO and GISMO-mis against independent constraint metrics

To assess whether GISMO and GISMO-mis are associated with different constraint scores, both metrics were correlated against other gene-level metrics. First, the GISMO metrics were compared against two human constraint metrics derived from gnomAD for loss-of-function constraint (LOEUF) and missense constraint (MOEUF). Next, to test whether GISMO and GISMO-mis can capture copy number variant dosage sensitivity, the scores were benchmarked against pTriplo (triplosensitivity) and pHaplo (haploinsufficiency)(22). We additionally benchmarked GISMO and GISMO-mis against gene-level AlphaMissense scores(27), which measures the effects of missense variation on predicted structural context from AlphaFold. A Spearman's correlation was used for all correlations.

### Partitioned heritability across independent traits

To assess the distribution of heritability enrichment across GISMO and GISMO-mis, LD score regression (LDSC) was used to partition heritability across gene deciles of both metrics(28–30). For both metrics, we included a 100kb flanking region both up and downstream for each gene and, in conjunction with genotype data from the 1000 Genomes Project. The SNPs were restricted

to HapMap3 SNPs with an estimated annotation-specific LD scores using a 1cM window. Next, partitioned heritability was applied to 276 independent traits from the UK Biobank (https://www.nealelab.is/uk-biobank/), as well as additional disease traits such as schizophrenia, bipolar disorder, autism spectrum disorder, attention-deficit hyperactivity disorder, and coronary artery disease(*31–35*). Phenotypes were selected from the UK Biobank based on having a significant p-value (P <0.05) after Bonferroni correction. Additionally, phenotypes were assessed for phenotypic correlation and only independent traits were included. The baseline model (v2.2), which includes 74 annotations to capture genomic properties, was included alongside the estimated LD scores as a predictor in the LD Score regression. HapMap3 SNPs, excluding the HLA region, were used as default regression SNPs.

### Assessing GISMO and GISMO-mis coding sequence biases

To assess whether GISMO and GISMO-mis are biased by coding sequence length, we correlated against the coding sequencing length (CDS) of each gene in both metrics. We compared against the gold standard for loss-of-function constraint, LOEUF from gnomAD. Given that LOEUF had a significantly stronger correlation with CDS relative to GISMO and GISMO-mis, we hypothesized that the GISMO metrics may help prioritize short essential genes. We considered genes with a LOEUF score <0.35 as LOEUF-constrained and intolerant, representing roughly the top 15% most constrained genes. Similarly, for both GISMO and GISMO-mis, we took the top 15% most constrained genes, which we considered intolerant. We compared the CDS length distribution for GISMO and GISMO-mis intolerant and LOEUF tolerant. We additionally looked at the inverse relationships to assess whether any constrained genes may be missed by GISMO and GISMO-mis.

## Analysis of LOEUF-constrained, GISMO-unconstrained X-linked genes

To examine divergence between human-specific and evolutionary constraint, we defined a discordant gene set consisting of genes in the top 15% most constrained by LOEUF and the bottom 20% of GISMO scores. Gene chromosome annotations were obtained from GENCODE v38, and the proportion of X-linked genes in the discordant set was evaluated relative to genome-wide distributions. To assess the functional relevance of these genes, we restricted the analysis to X chromosome genes only and intersected them with OMIM disease genes and the definitive recessive gene list from the Deciphering Developmental Disorders (DDD) study. Phenotype and pathway enrichment was performed using ToppGene, querying Human Phenotype Ontology (HPO) and curated pathway databases.

## Analysis of GISMO against gene family sizes

To determine if gene duplication and family size introduce technical bias into GISMO scores, an analysis was performed on the relationship between paralog counts and constraint metrics for all human protein-coding genes.

Gene family size was defined as the total number of paralogs present in the human genome for a given locus. Paralogous relationships and counts were extracted from Ensembl BioMart (Release 110/111) using the hsapiens_paralog_ensembl_gene attribute. Each gene was assigned a count representing the total number of its identified paralogs, which served as a proxy for the degree of duplication at that genomic position. To assess trends across different scales of duplication, genes were categorized into discrete bins based on their paralog count: 0 (single-copy genes), 1, 2, 3–5, 6–10, and 11–20. The analysis was capped at 20 paralogs to prioritize high-quality genomic

alignments and avoid the increased noise levels typical of exceptionally large or poorly assembled multicopy families. Distributional shifts were further examined using binned boxplots to monitor changes in the median and interquartile range (IQR) across categories.

Parallel stability analyses were performed for both GISMO scores and gnomAD LOEUF scores (v2.1.1) across the defined paralog bins. This comparison was intended to evaluate how the inter-species divergence signals utilized by GISMO, which are tracked through synteny-aware ortholog mapping (via TOGA), compare to the intra-species variation metrics used by LOEUF in duplicated regions.

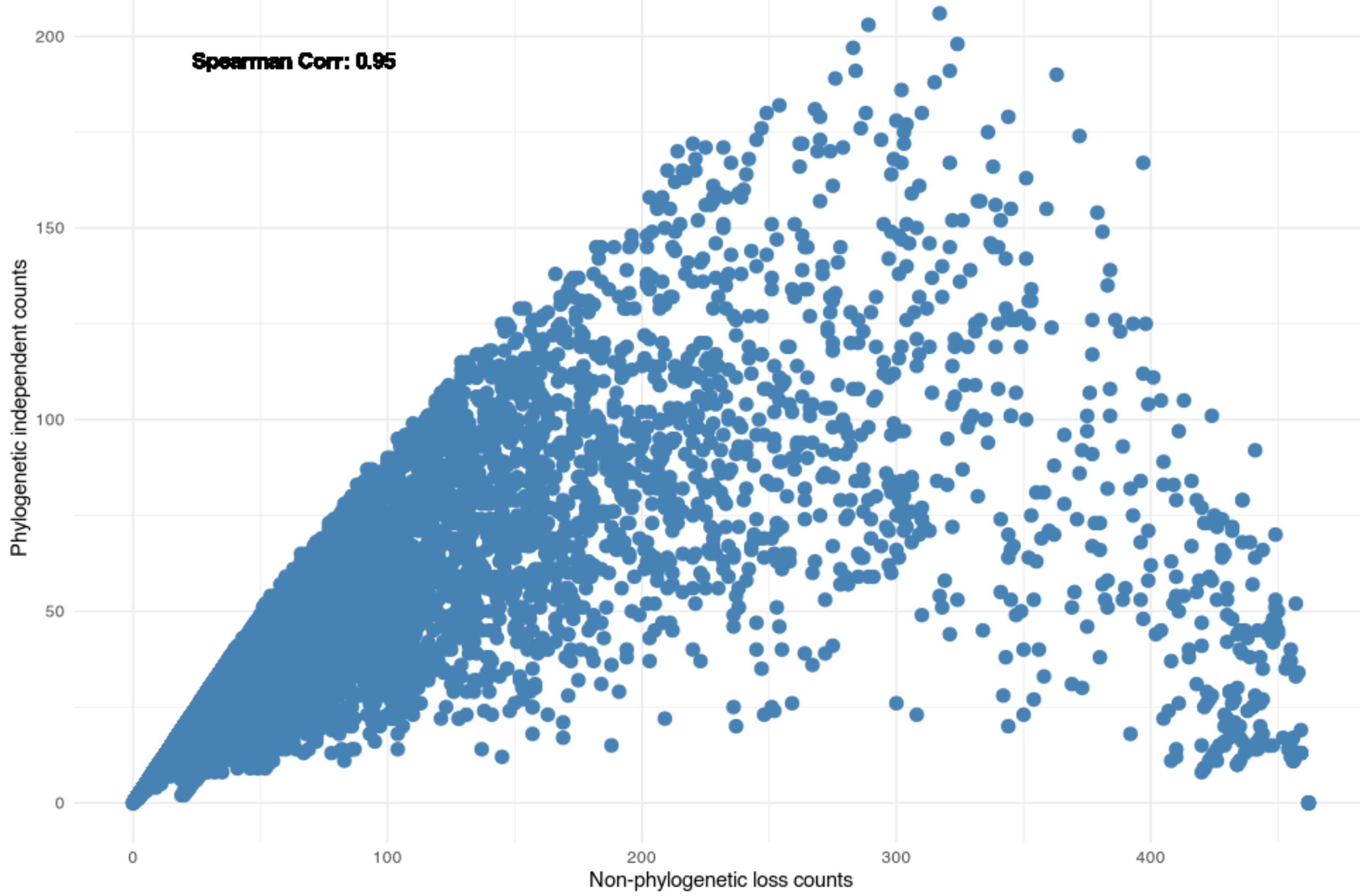


**Supplementary Figure 1. Relationship between phylogenetic independent counts and non-phylogenetic loss counts.** Each point represents a gene, with the x-axis indicating the number of non-phylogenetic loss counts and the y-axis indicating the number of phylogenetic independent counts. The Spearman correlation coefficient of 0.95, annotated in the top-left corner, signifies a strong positive association between the two variables.

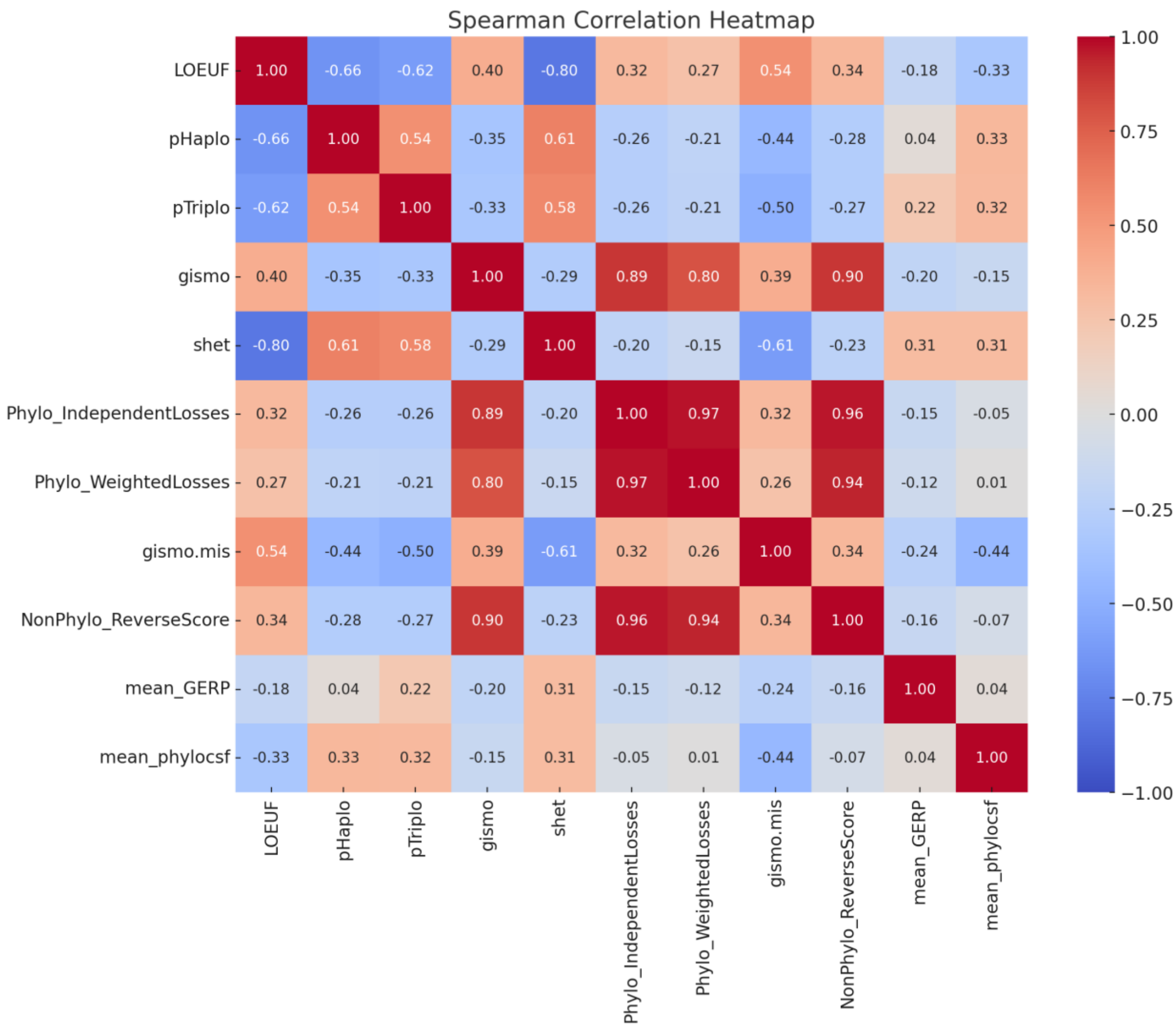


**Supplementary Figure 2.** Spearman Correlation Heatmap of Key Gene-Based Metrics. This heatmap shows pairwise Spearman correlation coefficients among LOEUF, pHaplo, pTriplo, gismo, sHet, Phylo_IndependentLosses (independent phylogenetic loss counts), Phylo_WeightedLosses (Independent phylogenetic loss counts weighted by evolutionary distance), gismo.mis, NonPhylo_ReverseScore (GISMO but with reverse weighting of evolutionary distance), gene level mean GERP scores, and mean PhyloCSF. Warmer colors indicate stronger positive correlations, while cooler colors indicate negative correlations.

The numerical values within each cell represent the Spearman correlation coefficient. Both rows and columns are hierarchically clustered to highlight groups of metrics with similar correlation patterns.

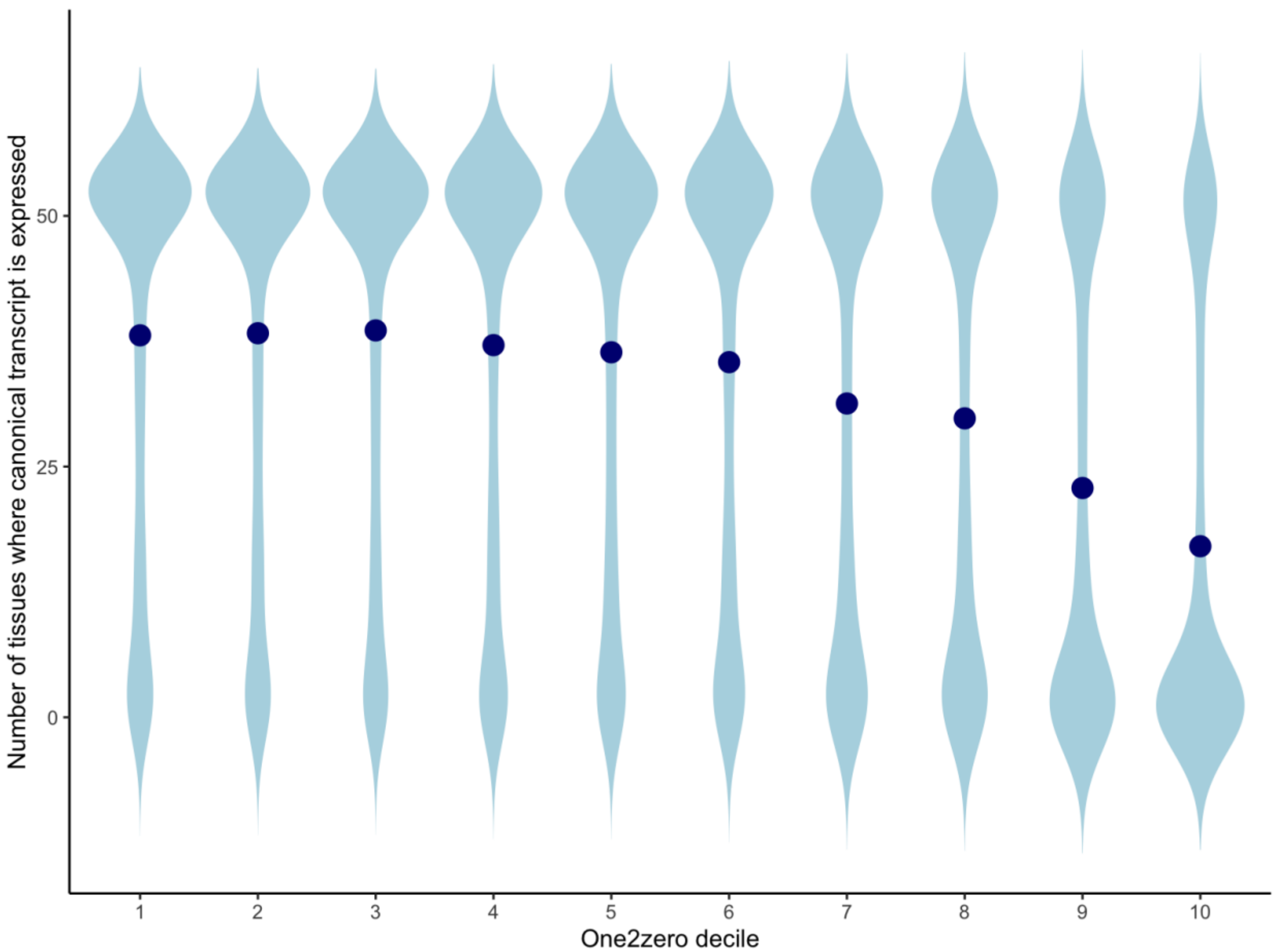


**Supplementary Figure 3. The most frequently lost genes across the mammalian tree tend to be expressed in the least number of tissues across GTEx 53.** The blue point represents the number of tissues that have any meaningful expression (>0.3 TPM) across 53 GTEx human tissues. One2zero is analogous to GISMO and represents a gene loss event, where a higher decile indicates highest gene loss.

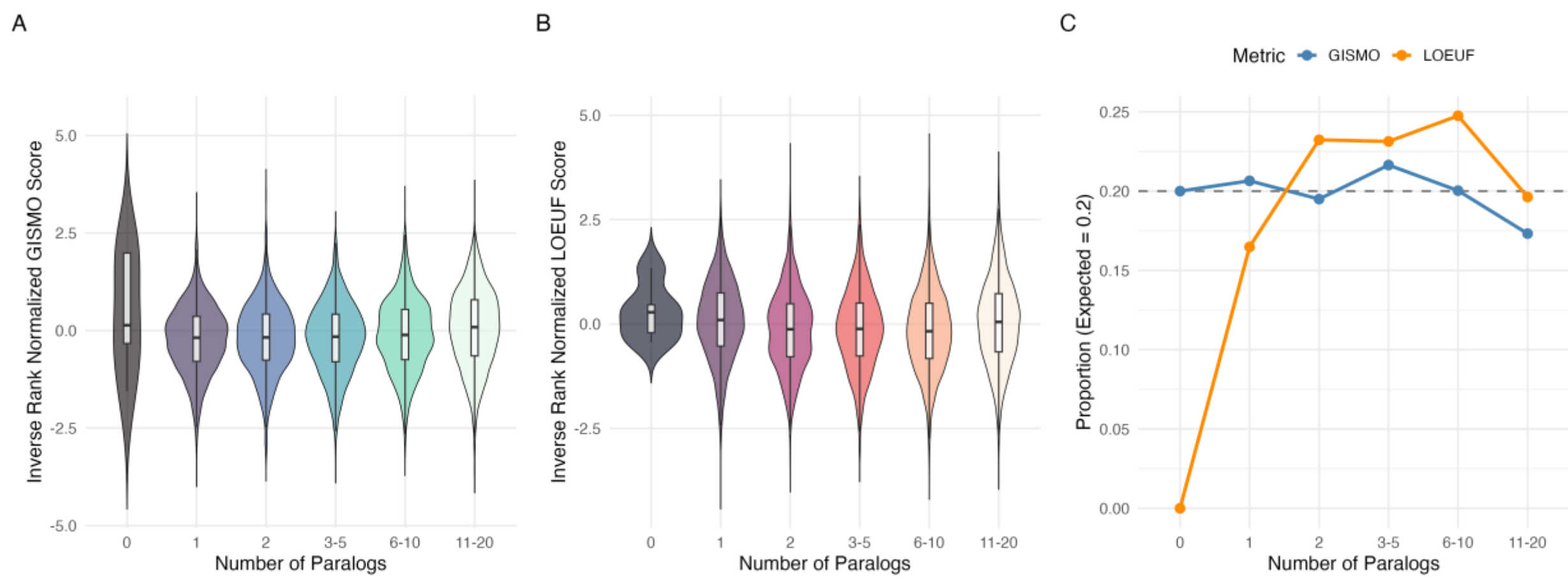


**Supplementary Figure 4: Evaluation of Constraint Metric Stability Across Gene Families. (A-B)** Violin plots illustrating the distribution of inverse rank-normalized scores for (A) GISMO and (B) LOEUF binned by the number of human paralogs. Lower scores indicate higher constraint (intolerance to loss). GISMO maintains a stable median and a broad distribution of constraint across all bins, whereas LOEUF shows a marked reduction in the identification of highly constrained genes (lower tails) as paralog count increases. **(C)** Proportion of genes identified within the top 20% of genomic constraint (bottom quintile) for each metric across paralog bins. The dashed horizontal line represents the genome-wide expected proportion (0.2). GISMO (blue) demonstrates consistent sensitivity across the spectrum of gene family sizes. In contrast, LOEUF (orange) shows a dramatic loss of sensitivity in non-duplicated and high-copy regions, likely reflecting technical limitations in short-read alignment within duplicated loci.

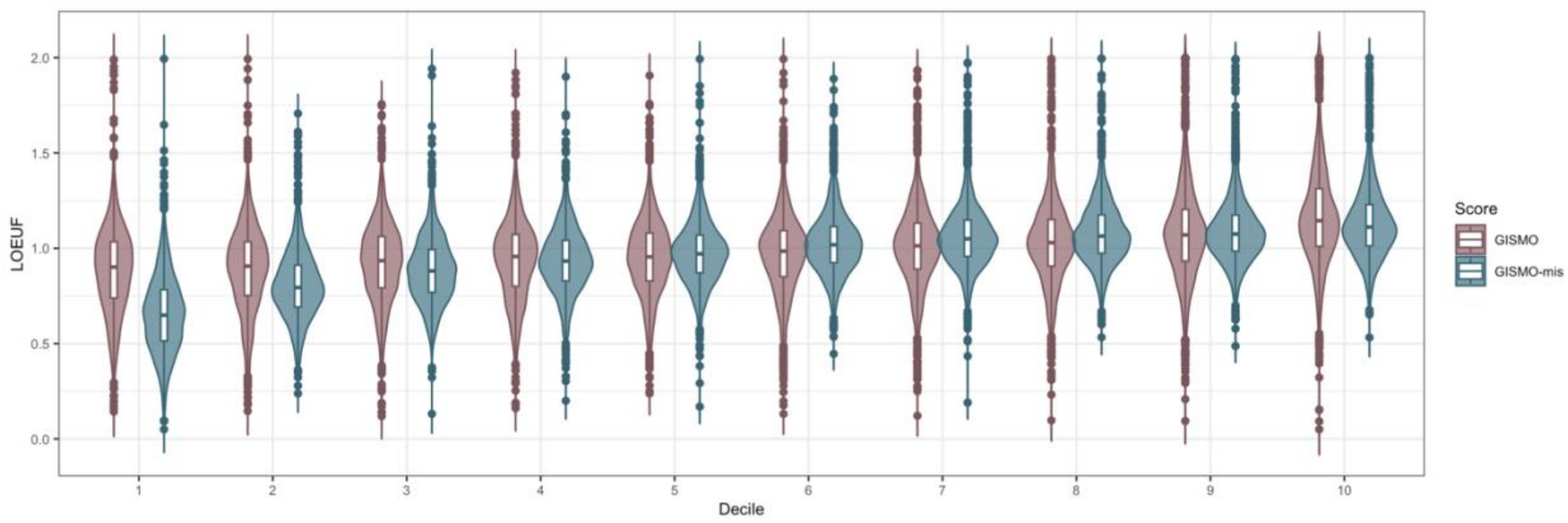


**Supplementary Figure 5. Comparison of GISMO and GISMO-mis to human missense constraint.** Missense constraint is defined as MOEUF from gnomAD, where lower deciles indicate most constrained.

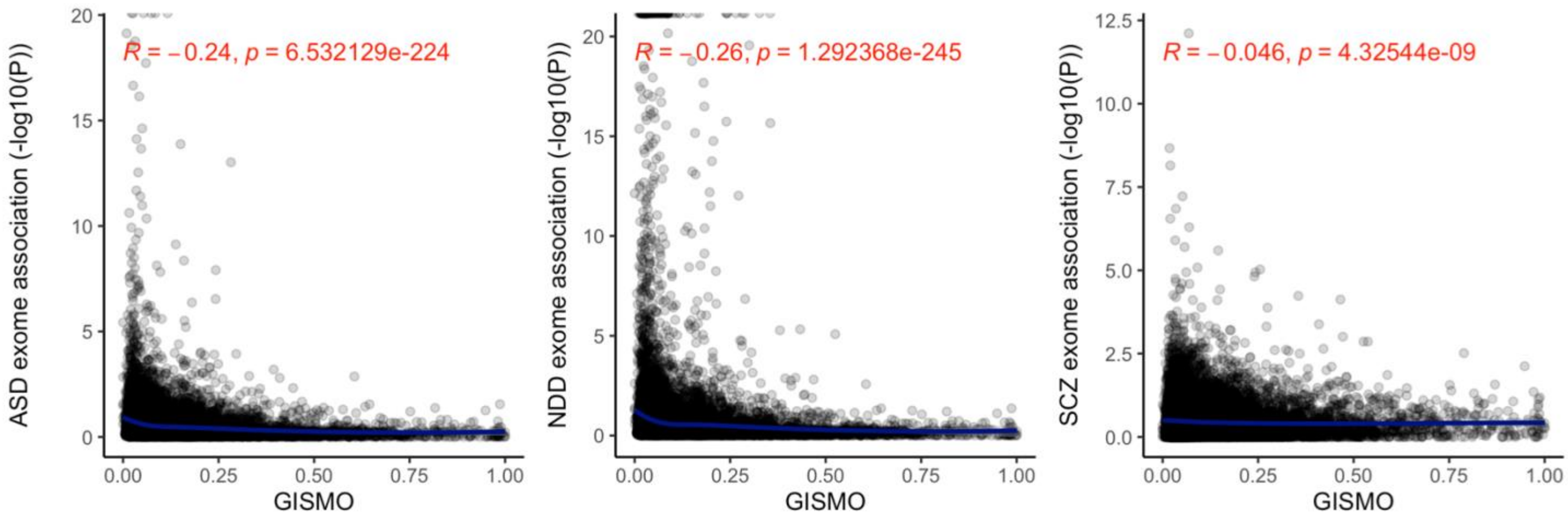


**Supplementary Figure 6. Association between neuropsychiatric rare variant associated genes and GISMO.** A Spearman's correlation was used.

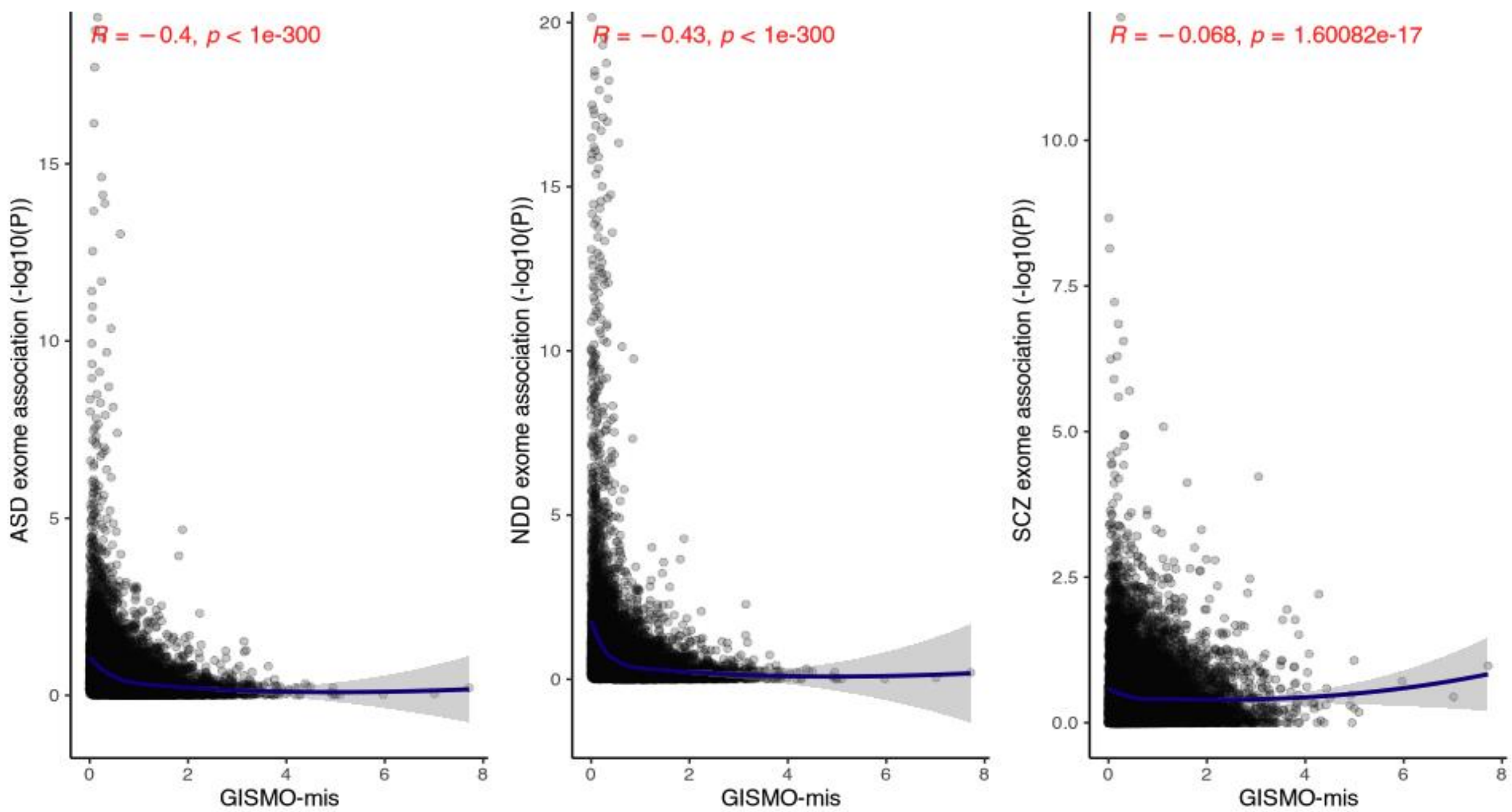


**Supplementary Figure 7. Association between neuropsychiatric rare variant associated genes and GISMO-mis.** A Spearman's correlation was used.

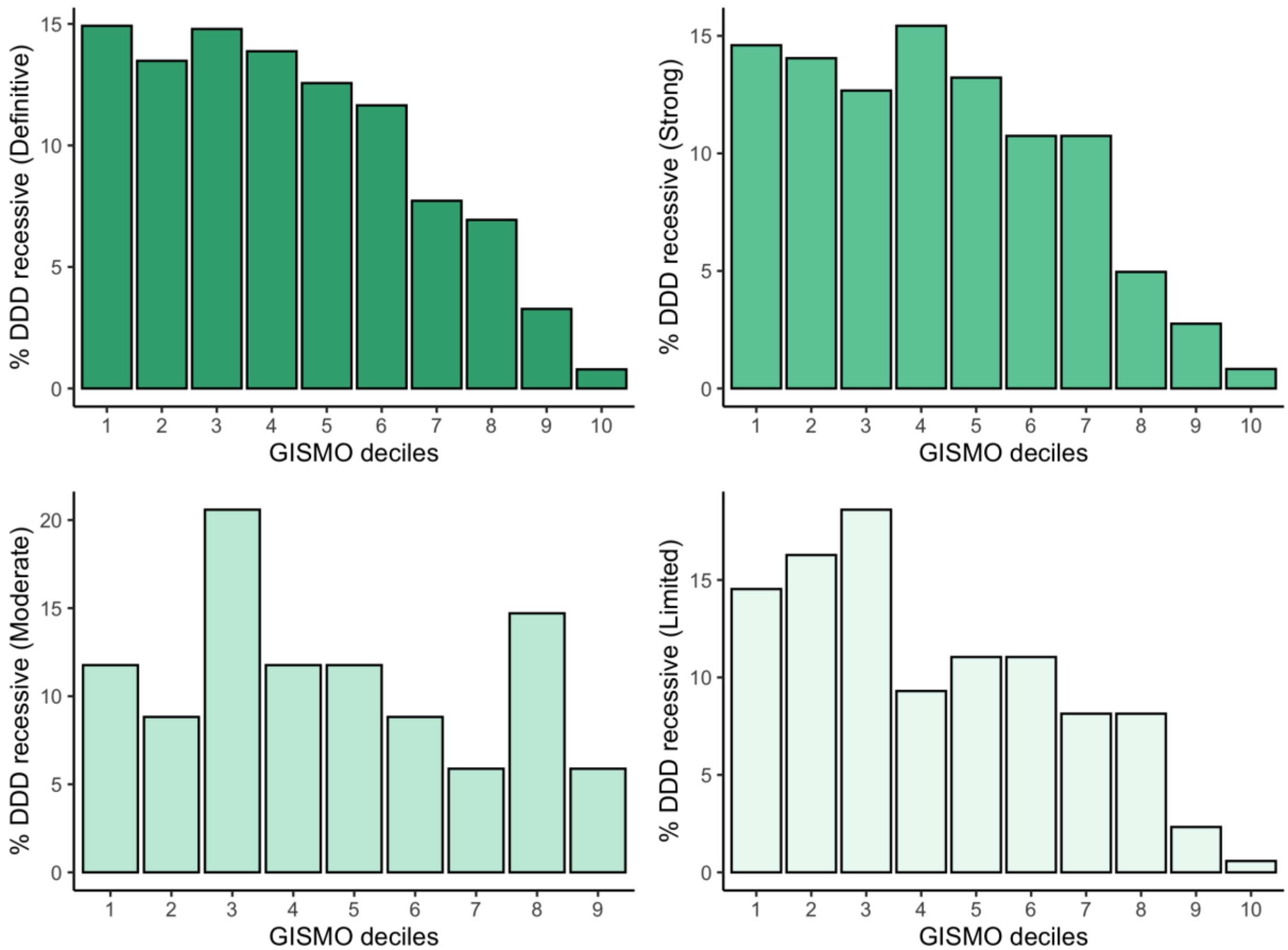


**Supplementary Figure 8. GISMO can help prioritize recessive disorder associated genes from the Deciphering Developmental Disorders (DDD) cohort.**
